# Modelling bone metastasis in spheroids to study cancer progression and screen cisplatin efficacy

**DOI:** 10.1111/cpr.13693

**Published:** 2024-06-20

**Authors:** Ceri‐Anne E. Suurmond, Sander C. G. Leeuwenburgh, Jeroen J. J. P. van den Beucken

**Affiliations:** ^1^ Dentistry—Regenerative Biomaterials, Radboudumc Nijmegen The Netherlands

## Abstract

Most bone metastases are caused by primary breast or prostate cancer cells settling in the bone microenvironment, affecting normal bone physiology and function and reducing 5‐year survival rates to 10% and 6%, respectively. To expedite clinical availability of novel and effective bone metastases treatments, reliable and predictive in vitro models are urgently required to screen for novel therapies as current in vitro 2D planar mono‐culture models do not accurately predict the clinical efficacy. We herein engineered a novel human in vitro 3D co‐culture model based on spheroids to study dynamic cellular quantities of (breast or prostate) cancer cells and human bone marrow stromal cells and screen chemotherapeutic efficacy and specificity of the common anticancer drug cisplatin. Bone metastatic spheroids (BMSs) were formed rapidly within 24 h, while the morphology of breast versus prostate cancer BMS differed in terms of size and circularity upon prolonged culture periods. Prestaining cell types prior to BMS formation enabled confocal imaging and quantitative image analysis of in‐spheroid cellular dynamics for up to 7 days of BMS culture. We found that cancer cells in BMS proliferated faster and were less susceptible to cisplatin treatment compared to 2D control cultures. Based on these findings and the versatility of our methodology, BMS represent a feasible 3D in vitro model for screening of new bone cancer metastases therapies.

## Graphical abstract

Bone metastatic spheroids were developed by co‐culturing healthy and cancer cells directly in ultra‐low attachment plates. Among other readouts, bone metastatic spheroids showed higher cancer cell proliferation and more relevant response to the chemotherapeutic cisplatin compared to 2D co‐cultures with similar seeding ratios.

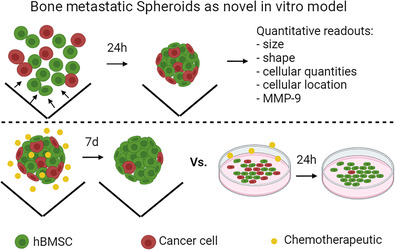

## 1 INTRODUCTION

Bone metastases are very painful cancer lesions caused by cancer cells that have metastasised from primary cancers, most often breast (70% metastasising to bone) or prostate (85% metastasising to bone) cancers.^1^ Once a breast or prostate cancer patient is diagnosed with bone metastases, 5‐year survival rates drop to 10% and 6%, respectively.^2^, ^3^ Cancer cells that cause bone metastases have some plasticity in the epithelial to mesenchymal transition to disseminate and subsequently extravasate into the bone microenvironment.^4^ This extravasation into bone is postulated to be facilitated by discontinuous endothelium in bone sinusoids, which partly explains the high incidence of metastases in bone compared to other organs.^5^ Subsequently, cancer cells in the bone microenvironment need to escape dormancy to mature as metastatic lesions.^4^ The bone microenvironment is a fertile soil for metastasis of cancer cells for several reasons. First, the haematopoietic environment, which normally nurtures haematopoietic stem cells, is hijacked by the plastic cancer cells. Second, the abundancy of growth factors in the bone microenvironment stimulates cancer cells, leading to the so‐called vicious cycle of bone metastases.^1^, ^5^, ^6^, ^7^ Based on the specific effects of cancer cells on various bone cells, bone metastases are either classified as osteolytic, osteoblastic or a combination thereof upon diagnosis using radiography. Furthermore, bone metastases can cause skeletal‐related events (SREs), such as pathological fractions, spinal cord compression and hypercalcemia. These SREs lead to a loss of mobility, decreased quality of life and substantial medical costs.^1^ Currently, multimodal treatment strategies are clinically applied, focusing on pain relief and restoration of function by combining metastatic tumour resection with either radiotherapy or chemotherapy. Such treatments can be fully curative for solitary metastases, especially in the long bones.^8^ However, for most patients, as evidenced by 5‐year survival rates, a curative outcome is not guaranteed while this treatment is associated with multiple drawbacks, including bone weakening following radiotherapy,^2^ challenging determination of cancer margins during lesion resection leading to cancer recurrence, and the creation of a critical sized bone defect after resection.^9^, ^10^ Furthermore, chemotherapeutic efficacy is strongly related to the type of primary tumour, while treatment efficacy differs considerably between patients, even for similar primary tumours causing metastases.^11^, ^12^, ^13^, ^14^, ^15^

To expedite clinical availability of effective bone metastasis treatments, reliable in vitro models for screening novel chemotherapeutics need to be developed,^16^ which preferably bear a potential for personalised medicine (e.g., by using cancer cells from patient biopsies).^17^ Such reliable models should include relevant human cell types, namely cancer cells that most frequently metastasise to bone (e.g., breast or prostate cancers cells) in combination with healthy non‐cancerous bone cells, to allow for clinically relevant assessment of chemotherapeutic specificity.^1^ For these healthy bone cells, primary human bone marrow stromal cells (hBMSCs) with mesenchymal stem cell (MSCs) markers are considered most relevant, as these cells are the precursors of differentiated osteoblasts and osteocytes.^18^, ^19^ Additionally, cancer cells are known to attract MSCs, resulting in relatively large numbers of MSCs at tumour sites.^20^ Furthermore, these models should rely on direct co‐cultures supporting cellular communication by both cell–cell contact and the secretome to resemble the physiological in vivo environment as closely as possible.^21^ Moreover, a predictive bone metastasis model should entail a 3D environment instead of the traditional 2D, since both MSCs and cancer cells behave differently in 3D cultures. For instance, cancer cells (including breast and prostate cancer cells) have demonstrated to be less susceptible to chemotherapeutic treatment in 3D cultures.^22^, ^23^, ^24^, ^25^, ^26^, ^27^, ^28^, ^29^ This reduced susceptibility corresponds more closely to outcomes obtained from in vivo studies, stressing the translational value of 3D cultures.^30^ Additionally, cancer cells in 3D cultures have different phenotypic, functional and metabolic behaviours, and demonstrate higher levels of malignancy, such as elevated levels of epithelial‐mesenchymal transition (EMT)‐associated proteins, and preservation of cancer stemness markers.^24^, ^26^, ^31^, ^32^ Next, MSCs in 3D cultures show lower proliferation rates, caused by an extended G0 phase of the cell cycle with higher levels of gene expressions related to self‐renewal.^33^, ^34^, ^35^ Finally, MSCs have shown higher osteogenic potential in 3D cultures.^36^

Currently, a sparse number of advanced 3D in vitro models for bone metastasis have been reported,^37^, ^38^, ^39^, ^40^, ^41^, ^42^, ^43^, ^44^, ^45^, ^46^, ^47^, ^48^, ^49^, ^50^, ^51^, ^52^, ^53^, ^54^, ^55^ which are formed by complicated and variable procedures, comprising one or multiple cell types in different configurations to reach near‐physiological conditions.^56^ Such models include on‐chip models, which use flow to mimic microvasculature. This allows to study cancer cell extravasation into the bone microenvironment.^37^, ^38^, ^39^, ^40^, ^41^, ^42^, ^43^, ^44^ Other examples include models based on different types of scaffolds, such as biomaterials^43^, ^44^, ^45^, ^46^, ^47^, ^48^, ^49^, ^50^, ^51^ and (decellularised) matrices produced by mesenchymal stem cells or osteoblasts in prolonged osteogenic culture conditions.^47^, ^52^, ^53^, ^54^, ^55^ These scaffold‐based approaches enable detailed studies on the process of bone metastasis, since cancer cells are strongly influenced by bone extracellular matrix (ECM)‐like materials. However, all of these bone metastasis models mainly focus on unravelling specific events within the bone metastasis process and are complicated to create, maintain, image and quantitatively characterise. Consequently, a relevant 3D model that allows for easy, fast and cheap screening of chemotherapeutic efficacy is still urgently needed.

Spheroids represent an emerging option to create a suitable 3D cell culture models, as these models are relatively easy to create, maintain, image and quantitatively characterise.^27^, ^31^, ^57^ Moreover, cells reside in a 3D environment in spheroids allowing for direct cell‐to‐cell contact. Furthermore, spheroids can be created without the use of biomaterials, which simplifies the interpretation of measured cellular responses,^58^ and increases the controllability and reproducibility of the model,^31^ which is of value for reliable in vitro models to be used for screening. Moreover, spheroids resemble the morphology of clinical tumours, which may partly explain the reduced susceptibility for chemotherapeutic treatment compared to planar cell cultures.^31^

We here developed a novel 3D bone metastasis model for in vitro evaluation of chemotherapeutic efficacy comprising a combination of human metastatic cancer cells and human MSCs in bone metastatic spheroids (BMS). The human cancer cell lines MDA‐MB‐231 and PC3 were deliberately selected since these are, metastasis originating, widely studied, and aggressive bone‐metastasising human cancer cell lines originating from breast and prostate cancer, respectively.^1^, ^59^, ^60^, ^61^ To the best of our knowledge, co‐culture spheroids composed of these specific cell types have not been previously created nor studied. BMS were optimised in terms of dimensions and cell ratios to achieve reliable spheroid generation and attain feasible quantitative analyses. Subsequently, BMS were characterised regarding spheroid morphology and cellular spatial organisation. Finally, BMS were directly compared to conventional planar co‐cultures with similar cellular seeding ratios with respect to their dynamic cell quantities during culture periods up to 7 days in the presence or absence of cisplatin.

## 2 MATERIALS AND METHODS

### 2.1 Cell culture procedures

hBMSC (passage: 2‐7), breast cancer cells (MDA‐MB‐231; ATCC) and prostate cancer cells (PC3; kindly provided by the Department of Urology, Radboudumc) were maintained in a log phase in a humidified atmosphere with 5% CO_2_ at 37°C with cell culture media composed of αMEM without ascorbic acid (A14090, Gibco) supplemented with 10% foetal bovine serum (Gibco) and 1% penicillin–streptomycin (Gibco).

hBMSCs were isolated from iliac bone fragments from healthy donors after surgery (Department of Maxillofacial Surgery, Radboudumc, The Netherlands) after ethical approval (Commissie Mensgebonden Onderzoek: dossier number #2017‐3252) as described previously.^62^ In line with the criteria as set by the International Society for Cellular Therapy (ISCT),^63^ hBMSCs immunophenotypically expressed characteristic MSC markers (>95% immunopositive for CD73, CD90 and CD105, and immunonegative for CD45) and the capacity to undergo osteogenic differentiation.

### 2.2 Spheroid formation

To optimise spheroid formation (*n* = 4), hBMSC/cancer cell combinations were seeded in ultra‐low attachment plates (ULA plates, PHCBI) at different total cell numbers (range: 2000–6000 cells per 200 μL) and different hBMSC/cancer cell ratios (9:1 and 1:1). Methylcellulose (MC; Sigma Aldrich; 0.24, 1.2 mg/mL), collagen type 1 from rat tail (Corning; 30 μg/mL) or a combination thereof were used as additives promoting spheroid formation. At days 1, 3 and 7 days of culture, media was carefully refreshed.

### 2.3 Quantitative characterisation of bone metastasis spheroids

Spheroid morphology (*n* = 5) was characterised via imaging at 5× magnification at multiple timepoints using a brightfield microscope (Leica DM IL LED) and subsequent analyses using image analysis protocols in FIJI^64^ (see Supplementary Information 1.1 for further details), to assess spheroids projected area, diameter and circularity.

Spheroid total DNA content (*n* = 23) was characterised by a Quantifluor DNA assay (Promega) with adjusted cell lysis buffer as described previously.^65^

### 2.4 Cellular quantities in 2D planar co‐cultures

Cellular quantities and ratios in 2D planar co‐cultures (*n* = 3) were determined based on CellTrace staining (ThermoFisher Scientific; Far Red for cancer cells and CFSE green for hBMSCs) and subsequent cell seeding in 8‐well microwell slides (ThermoFisher Scientific) at a cell density of 15,000 cells/cm^2^. On each timepoint, cells were counterstained with 2 μg/mL Hoechst 33342 (ThermoFisher Scientific) for 20 min and fixated with 10% formalin. For fluorescence imaging (Zeiss Axio Imager Z2 fluorescence microscope at 10× magnification), nine pictures per well were stitched and subsequently analysed (see Supplementary Information 1.2).

### 2.5 Cellular quantities and spatial organisation in 3D bone metastasis spheroids

Cellular quantities, ratios and spatial organisation in 3D spheroid co‐cultures (*n* = 3) were assessed based on confocal laser scanning microscopy images (Carl Zeiss LSM880) images. Before imaging, spheroids were counterstained with 2 μg/mL Hoechst 33342 (ThermoFisher Scientific) for 40 min, then resuspended in Optimem (Gibco) and transferred to glass bottom microwell slides (Ibidi) for live imaging. Spheroid halves were imaged via z‐stacks at 20× magnification (z‐step depth: 1.48 μm) and analysed using FIJI^66^ (see Supplementary Information 1.3).

Simplification of the imaging and quantification processes was implemented via validation of representativeness of a single slice (at 25% depth of the whole spheroid; see Supplementary Information 1.5), which showed reliable similarity (see Supplementary Information, Figure S1) which was in line with previously reported studies.^67^ Similarly, the spatial organisation of the cancer cells and hBMSCs in the spheroids was characterised using FIJI by setting a core (circular area with half of the diameter of the total spheroid) and periphery (total spheroid minus core) selection, which was then quantified in terms of total area for each cell type (see Supplementary Information 1.4).

### 2.6 Cisplatin susceptibility assessment

3D spheroids (*n* = 3) and 2D planar co‐cultures (*n* = 2, seeding cell density of 7500 cells/cm^2^), 24 h after cell seeding, were exposed to various concentrations (10, 25, 50 and 100 μM) of the chemotherapeutic model drug cisplatin for 24 h (AK scientific) after which cells were thoroughly washed with PBS and supplemented with fresh media. On days 1, 3 and 7 after cisplatin addition the cells were quantitatively analysed (see Sections 2.4 & 2.5).

Furthermore, spheroids (*n* = 5) were evaluated for total ATP content with a 3D CellTiterGlo assay (Promega) according to manufacturer's instructions and normalised against untreated controls per time point.

### 2.7 MMP‐9 measurements in supernatant

Supernatant of 3D spheroids (*n* = 3) after cisplatin exposure (0 and 50 μM) was collected on days 1, 3 and 7, and stored at −70°C until measurements. Matrix metalloproteinase 9 (MMP‐9) was measured with an ELISA (Biotechne, R&D systems) according to manufacturer's instructions.

### 2.8 Statistics

Data are expressed as mean value ± standard deviation, with individual data points in case of bar graphs. Statistical analysis was performed using Prism software (GraphPad, version 8; La Jolla California USA). Multiple independent *t*‐tests were performed for quantitative bone metastasis spheroid morphology characterisation data and cellular spatial localisation data (for each location) on each time point. One‐way ANOVA followed by Tukey multiple comparison post‐hoc tests were performed on the DNA characterisation data and ATP data after cisplatin treatment for both the breast and prostate cancer conditions. Multiple independent *t*‐tests were performed comparing the (relative) cellular quantities of the hBMSCs and cancer cells in spheroids and planar co‐cultures on the different timepoints. Additionally, a one‐way ANOVA followed by Tukey multiple post‐hoc tests was performed to analyse (relative) cancer cell quantities in spheroids and planar co‐cultures. To compare relative cancer cell quantities in planar cultures and spheroids, Student's *t*‐tests were performed at multiple time points. Multiple independent *t*‐tests were performed for comparing of MMP‐9 levels in the supernatant of the BMS. For all statistical analyses, a *p*‐value <0.05 was considered significant.

## 3 RESULTS AND DISCUSSION

### 3.1 Creation and characterisation of BMS model

To create co‐culture 3D spheroids with near‐physiological resemblance of bone metastases, we combined healthy hBMSCs and cancerous cells at various cell culture conditions in ultra‐low attachment plates. These plates facilitate upscaling and straightforward creation of stable spheroids.^68^ Importantly, previous work demonstrated that cancer cell spheroids created in this platform showed strong similarity to cancer cell spheroids produced using more traditional hanging drop and spinning flasks techniques.^69^ To optimise spheroid formation, we assessed spheroid shape and stability as a function of (1) addition of medium additives, (2) variation of hBMSC:cancer cell ratio and (3) total cell number per spheroid.

To support the formation of spheroids, we added either methylcellulose (0.24 or 1.2 mg/mL), collagen (30 μg/mL), or a combination thereof to the medium in which the spheroids composed of hBMSCs, MDA‐MB‐231 or PC3 cells were cultured (Figure S2A), since previous work showed that spheroid circularity is enhanced by supplementation of these additives.^70^, ^71^ Although the mechanism of the spheroid‐supporting effect of methylcellulose remains unclear, it has been postulated that methylcellulose functions as a molecular crowder decreasing the intercellular distance.^70^ Alternatively, collagen fibrils supplemented to the media can interact with cells, thereby enhancing cell–cell contact required for spheroid formation.^71^ Since we observed that spheroid formation was most rapid and stable at highest circularity in the presence of 30 μg/mL of collagen supplemented to the medium (Table S1), we selected this supplementation type and concentration for further studies.

We next explored stable spheroid formation of hBMSCs and cancer cells at different cell ratios. Based on pilot studies, spheroids with a 9:1 (hBMSC:cancer cell) ratio were chosen as this ratio demonstrated a vast increase of cancer cells within 3 days of culture, while remaining stable; higher cancer cell ratios (e.g., 1:1) rapidly became irregular in shape due to very high proliferation of cancer cells (Figure S2B).

To optimise spheroid size with respect to stability and imaging feasibility, we varied total cell seeding number per spheroid from 2000 to 6000 cells. This range was based on the possibility of containing a physiological gradient without containing a necrotic core, as previous literature reported that hBMSC spheroids composed of 3000 cells contained hypoxia markers,^72^ while dead cells were not reported in spheroids up to 60,000 cells.^73^ On the other hand, most cancer spheroids developed a necrotic core when comprising >4000 cells.^74^, ^75^ Based on brightfield images recorded on day 3 of cell culture (Figure S2C) combined with FIJI analysis of spheroid circularity (Table S2), spheroids comprising 2000 cells demonstrated optimal stability and circularity, while remaining sufficiently small to enable facile processing for imaging. Summarising, based on the above‐described optimisation, we selected 30 μg/mL collagen as a medium additive, a hBMSC:cancer cell ratio of 9:1, and an initial number of 2000 cells per spheroid for further BMS experiments.

It should be noted that our BMS model does not fully capture the full complexity of the bone metastasis process characterised by various processes such as extravasation, intravasation, dormancy and activation as well as the presence of multiple cell types, the extracellular matrix and mechanical loading.^56^, ^76^, ^77^ We deliberately simplified our model since overcomplexity inevitably impedes sensitive readouts. Previous bone metastasis in vitro models also included only few factors to further unravel key events in bone metastasis.^37^, ^38^, ^39^, ^40^, ^41^, ^42^, ^43^, ^44^, ^45^, ^46^, ^47^, ^48^, ^49^, ^50^, ^51^, ^52^, ^53^, ^54^, ^55^ However, the key factors we selected for a relevant model of bone metastasis for in vitro evaluation of chemotherapeutic efficacy are 3D culturing and co‐culturing of multiple cell types, since they allow for highly relevant cell–cell interactions and resulting relevant cellular behaviours.^31^, ^56^, ^76^, ^77^, ^78^

**FIGURE 1:**
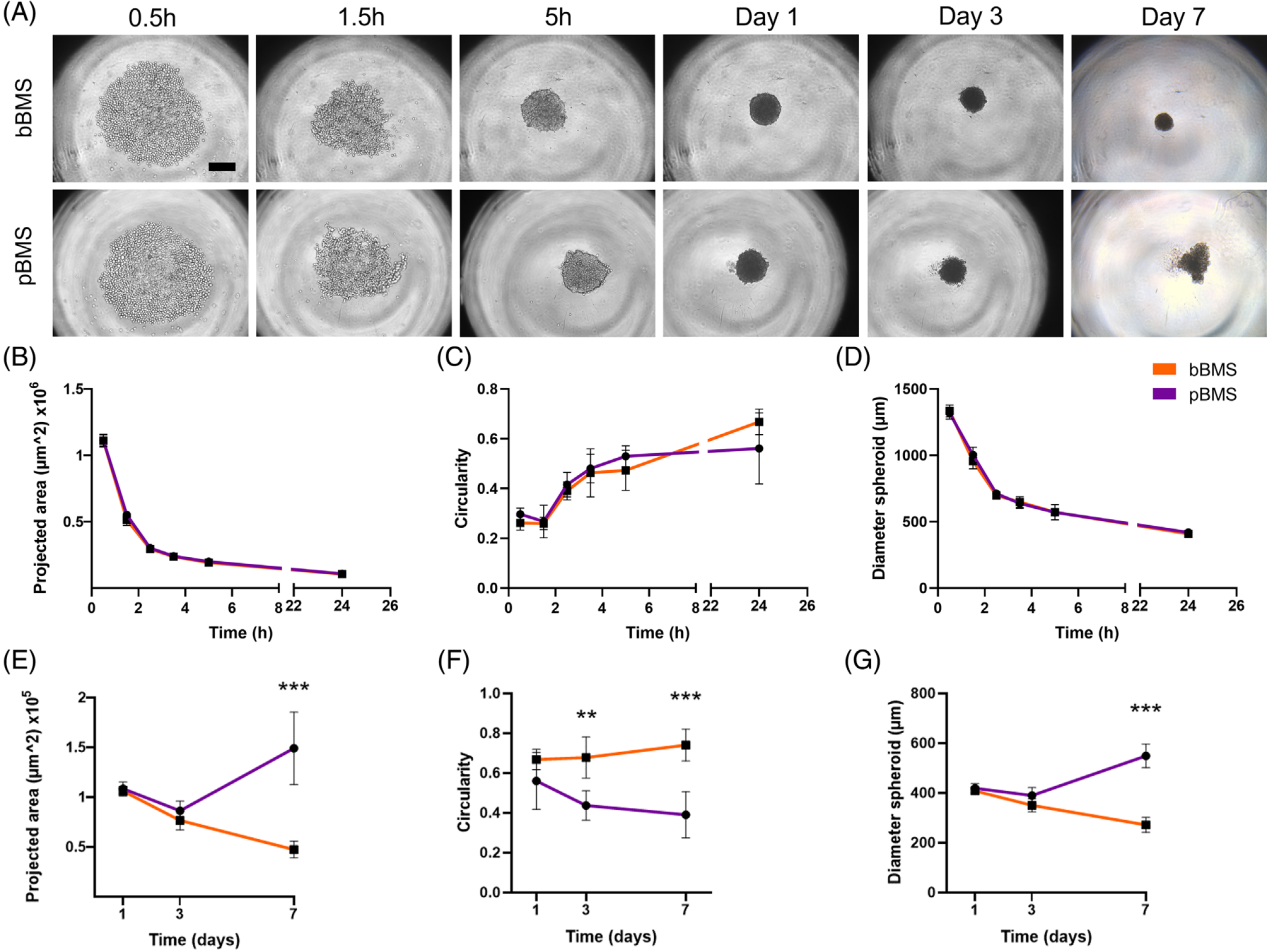
Bone metastasis spheroid formation with breast or prostate cancer cells. Brightfield images (A) show spheroid formation within 24 h. Spheroid formation was accompanied by a decrease in projected area (B), increase in circularity (C) and decrease in spheroid diameter (D). Prolonged culture periods up to 7 days demonstrated differences between bBMSs and pBMSs regarding projected area (E), circularity (F) and increased diameter (G). Quantitative data are based on spheroids (*n* = 5) and were statistically analysed using an independent *t*‐tests at each timepoint; *p < 0.05; **p < 0.01; ***p < 0.001. Scale bar represents 300 μm. bBMS, breast BMS; BMS, bone metastasis spheroid; pBMS, prostate cancer BMS.

Using our above‐described optimised spheroid formation protocol, we monitored BMS morphology using brightfield imaging during formation (up to 24 h) and prolonged culture periods (up to 7 days; Figure 1A), for both BMSs comprising either breast (bBMS) or prostate cancer (pBMS) cells. The morphology of both bBMSs and pBMSs remained largely similar during spheroid formation in terms of projected area (Figure 1B), circularity (Figure 1C) and diameter (Figure 1D). With increasing culture time, both bBMSs and pBMSs became more compact (to about ~400 μm which is one third of their original diameter) and circular. These diameters correspond to previously reported values for spheroids after 24 h composed of either hBMSCs, MDA‐MB‐231 or PC3 cells.^70^, ^79^ During prolonged culture periods up to 7 days, we observed apparent differences between bBMSs and pBMSs (Figure 1E–G). Whereas bBMSs continued compaction combined with increased circularity, pBMSs became morphologically irregular leading to significantly lower circularity values (*p* < 0.05) and increasing spheroid size (*p* < 0.05). Likely, both hBMSCs and cancer cells contribute to the size and shape of BMS over 7 days of culture as spheroids of MDA‐MB‐231 cells were reported to increase in size,^80^, ^81^ while spheroids of hBMSCs decrease in size over longer culture times.^79^

Furthermore, the spheroid size could not be directly related to the number of cells present in BMS, as total cellular DNA per spheroid remained very similar over time (Figure S3), which was also previously reported for spheroids composed of osteoblasts or hBMSCs.^36^, ^82^ This is in contrast to typical planar cultures, in which most cell types expand exponentially.

### 3.2 Relative hBMSC and cancer cell quantities evolve differently in planar co‐cultures versus BMSs

**FIGURE 2:**
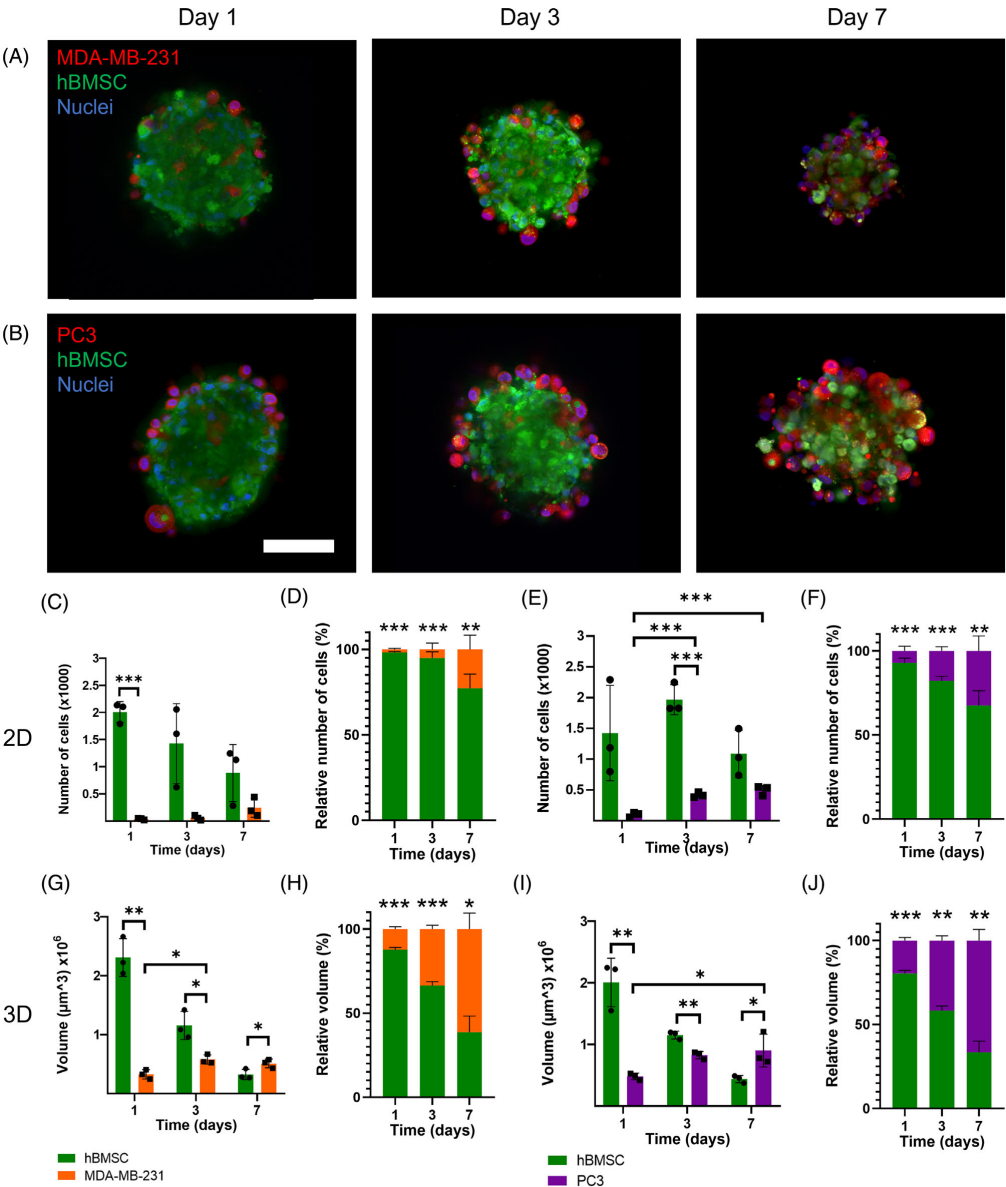
Quantities of hBMSCs and breast or prostate cancer cells in planar co‐cultures and spheroids over time. Fluorescence images of bBMS (A) and pBMS (B) enabled quantification. Planar cultures show only a minimal cancer cell growth for both the cell number (C & E) and relative cell number (D & F), while in BMSs differences of cellular volumes between hBMSCs and cancer cells (G & I) diminish over time, with high relative cellular volumes of cancer cells (H & J) on day 7. Quantitative data of BMSs and planar cultures are based on spheroids (*n* = 3) or wells (*n* = 3) and were statistically analysed using one‐way ANOVA with Tukey multiple comparison post hoc test; *p < 0.05 for the cancer cells over time, and per time point with an independent *t*‐test on the different cell types; *p < 0.05; **p < 0.01; ***p < 0.001. Scalebar represents 300 μm. hBMSCs are coloured green, cancer cells red and nuclei blue. bBMS, breast BMS; BMS, bone metastasis spheroid; hBMSCs, human bone marrow stromal cells; pBMS, prostate cancer BMS.

To compare the relative number of hBMSC versus cancer cells in both planar co‐cultures and BMSs over time, cells pre‐stained with fluorescent labels (green for hBMSCs and red for cancer cells) were imaged at days 1, 3 and 7 (Figure 2A,B; Figure S4A,B). Halves of BMSs were imaged with z‐stacks, which were then quantified in FIJI for total cell volume for each cell type. For this binary z‐stacks were created based on the fluorescence signal for each cell type after proper thresholding. Next, using the z‐step length, the total cellular volumes were calculated in FIJI 3D suite.^66^ Fluorescence images of the planar cultures were quantified by counting the number of nuclei for each cell type. Subsequently, for both models relative cellular quantities were calculated to allow for direct comparison between planar co‐cultures and BMSs. Live cells were imaged in BMSs as this procedure reduces the chance of sample artefacts.^83^

Both the breast and prostate planar co‐cultures contained limited numbers of cancer cells on day 1, that is, 2% and 7% of the total cellular population, respectively, which slightly but significantly increased to 23% and 33% (both *p* < 0.01 comparing day 1 vs. day 7) at day 7 (Figure S4A,B; Figure 2C–F). Morphologically, breast cancer cells on day 7 were evenly distributed between the elongated and stretched hBMSCs, which corroborates findings reported by Mandel et al. who reported proliferation of MDA‐MB‐231 cells in presence of MSCs with similar morphologies for both cell types.^84^ Planar co‐cultures with prostate cancer cells showed a different morphology for the prostate cancer cells, appearing as islets surrounded by hBMSCs; few relative large cancer cells were observed on day 7, which suggests cancer cell stemness.^85^ High proliferation and a well‐organised structure of prostate cancer cells in co‐culture with stem cells were also observed by Li et al.^86^ Although they observed a well‐organised structure, no apparent islets of cancer cells were observed. However, in another tri‐culture of MSCs, endothelial cells and PC3 cells, in which the healthy cells were pre‐cultured before cancer cell seeding, this islet structure was reported as well.^87^

Cancer cells in bBMSs and pBMSs likely proliferated faster than in planar co‐cultures (Figure 2A,B,G–J). On day 1, BMSs comprised 12% and 19% breast and prostate cancer cells, respectively, which significantly increased to 61% and 67% at day 7 (both *p* < 0.001 comparing day 1 vs. day 7 of bBMSs and pBMSs, and *p* < 0.01 comparing planar co‐cultures with BMSs on day 7). Besides cancer cell proliferation, the relative dominance of cancer cells in BMSs may be partly explained by slowly proliferating hBMSCs with an accompanying decrease of their cellular volumes, as MSCs usually become more quiescent in 3D cultures, which is typical for their in vivo niche,^34^, ^35^, ^73^ while also decreasing in size in other 3D cultures.^79^ Green coloured vacuoles on days 3 and 7 in cancer cells (Figure S5), which could be described as cancer cell cannibalism^88^, ^89^ could effectuate the higher cell proliferation and survival rates of cancer cells.^88^ The relative increase of cancer cells in the BMSs seems to contradict previous work where breast and prostate cancer cells became less proliferative in 3D cultures.^24^, ^25^, ^26^, ^29^ However, cell behaviour in BMSs was not only affected by the three dimensionality of this model, but also by the direct cell–cell contact with the hBMSCs.

To the best of our knowledge, no similar co‐culture spheroids composed of hBMSCs and MDA‐MB‐231 or PC3 cells have been previously reported, but other 3D direct co‐cultures of MDA‐MB‐231 or PC3 cells with healthy cells (hBMSCs differentiated into osteoblasts,^46^ hBMSCs,^51^ a human foetal osteoblast cell line,^88^ or osteoblasts^58^) also reported a relative fast proliferation of cancer cells. Furthermore, other breast and prostate cancer cell lines were reported to proliferate when co‐cultured with hBMSCs^50^ or MSCs with endothelial cells.^87^ In contrast, other types of healthy cells have shown to inhibit cancer cell proliferation. For example, Marlow et al. reported that a quadruple‐culture of human foetal osteoblasts, a mesenchymal immortalised cell line, and primary human umbilical vein endothelial cells inhibited proliferation of multiple breast cancer cell lines.^50^ This decreased cell proliferation was also observed for prostate cancer cells in co‐culture with osteoblasts^90^ or in a tri‐culture of osteoblasts and endothelial cells.^32^ This inhibiting effect of these differentiated cells on cancer cell proliferation was also observed for MSCs, which were reported to inhibit cancer cells in various types of in vitro systems by inducing cancer cell apoptosis, triggering cancer cell cycle arrest or reducing invasion capabilities of cancer cells.^91^, ^92^, ^93^ Although we mainly included hBMSCs in our BMS models to allow for testing of chemotherapeutic specificity, it can clearly be concluded that the stem cells in our model support cancer cell proliferation.

To further fine‐tune our BMS model, other cell types of the bone environment could be included, such as osteocytes (with an important role in bone homeostasis^23^, ^89^), fibroblasts (as a predominant cell type in metastatic cancer lesions^90^), immune cells (for evaluating immunotherapies^80^) or osteoclasts (responsible for bone resorption^94^ and with a crucial role in the bone pain associated with bone metastases^1^, ^95^).

**FIGURE 3:**
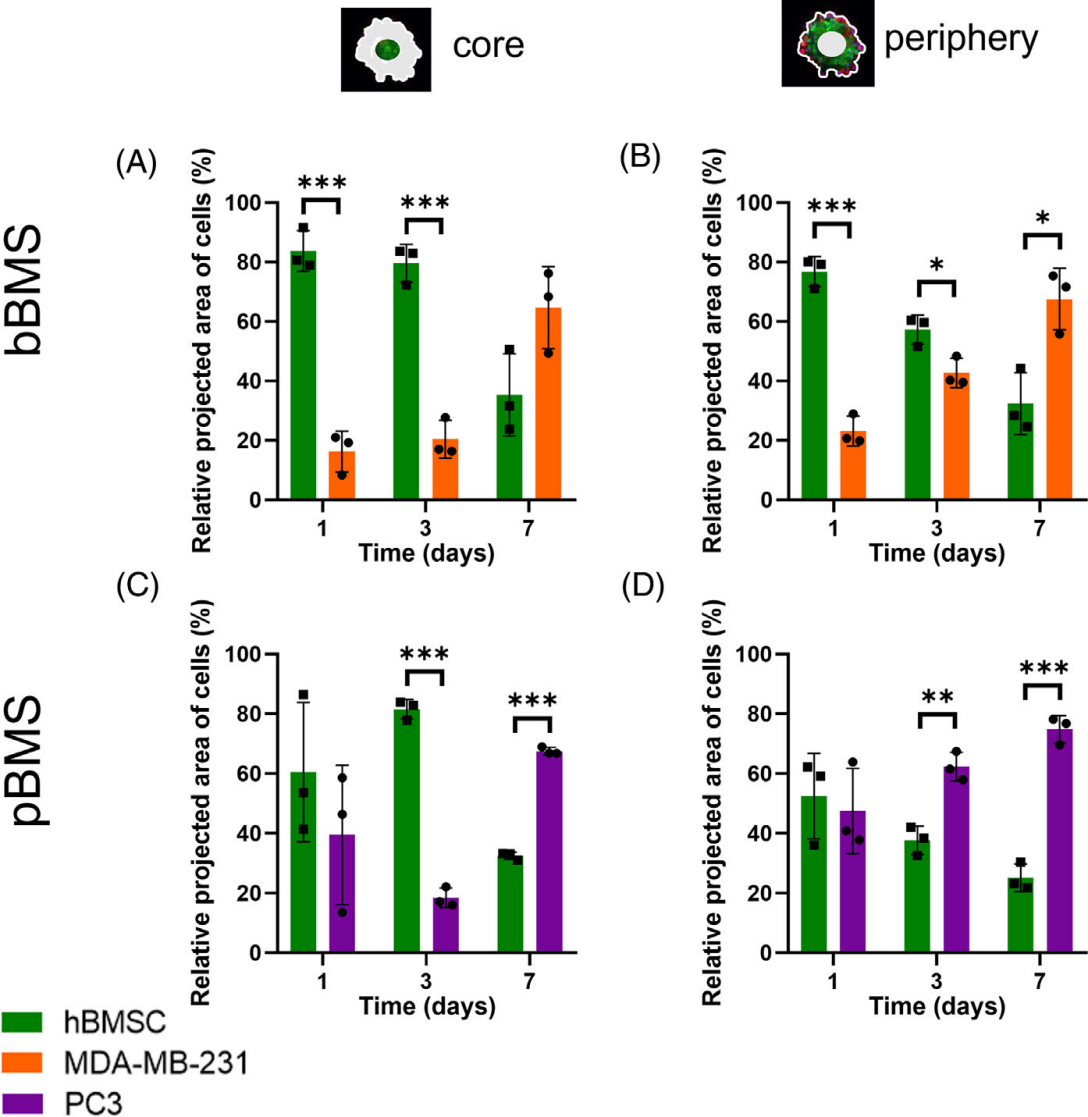
Cellular spatial localisation within BMSs. Both in the core (A, C) and in the periphery (B, D) of BMSs, cancer cells became the dominant cell type after 7 days of culture. Quantitative data of the BMSs (*n* = 3) were statistically analysed using an independent *t*‐test on each timepoint comparing the different cell types; *p < 0.05; **p < 0.01; ***p < 0.001. BMS, bone metastasis spheroid.

Besides cancer cell proliferation within BMSs, we also observed a heterogeneous cellular spatial organisation within these BMSs, as cancer cells were predominantly located around a core of hBMSCs on day 3 (Figure 2A,B). In standardised core (Figure 3A,C) and periphery (Figure 3B,D) selections of BMSs, the total cell areas for hBMSCs and cancer cells were determined. These quantitative data confirmed that hBMSCs and cancer cells were mainly present in the core versus periphery, of both bBMSs and pBMSs. This type of cellular spatial organisation was also previously observed for co‐culture spheroids of MSCs with either C42B prostate cancer cells or B‐Cell non‐Hodgkin lymphoma cells.^96^, ^97^ The differential adhesion hypothesis seems to be a plausible explanation for this cellular organisation, as it considers cells as liquids containing different surface adhesions by which they organise themselves in a heterogenous population to minimise their interfacial free energy. Here, cells with a lower surface tension tend to envelop cells with a higher surface tension. Surface adhesions are linked to different levels of cadherin expression, which are expressed more abundantly on hBMSCs than on metastasising MDA‐MB‐231 and PC3 cancer cells.^98^, ^99^, ^100^ This envelopment only occurs above a critical ratio, which might explain why this heterogeneous cellular spatial organisation only occurred on day 3 when cancer cells proliferated sufficiently to reach this ratio. However, further cancer cell proliferation towards day 7 evoked loss of this cellular spatial organisation, because cancer cells became the dominant cell type independent of their location within the BMS. However, the cellular distribution occurring here might not be typical behaviour as would be seen in in vivo models.

### 3.3 Planar co‐cultures respond differently to cisplatin treatment compared to BMSs

**FIGURE 4:**
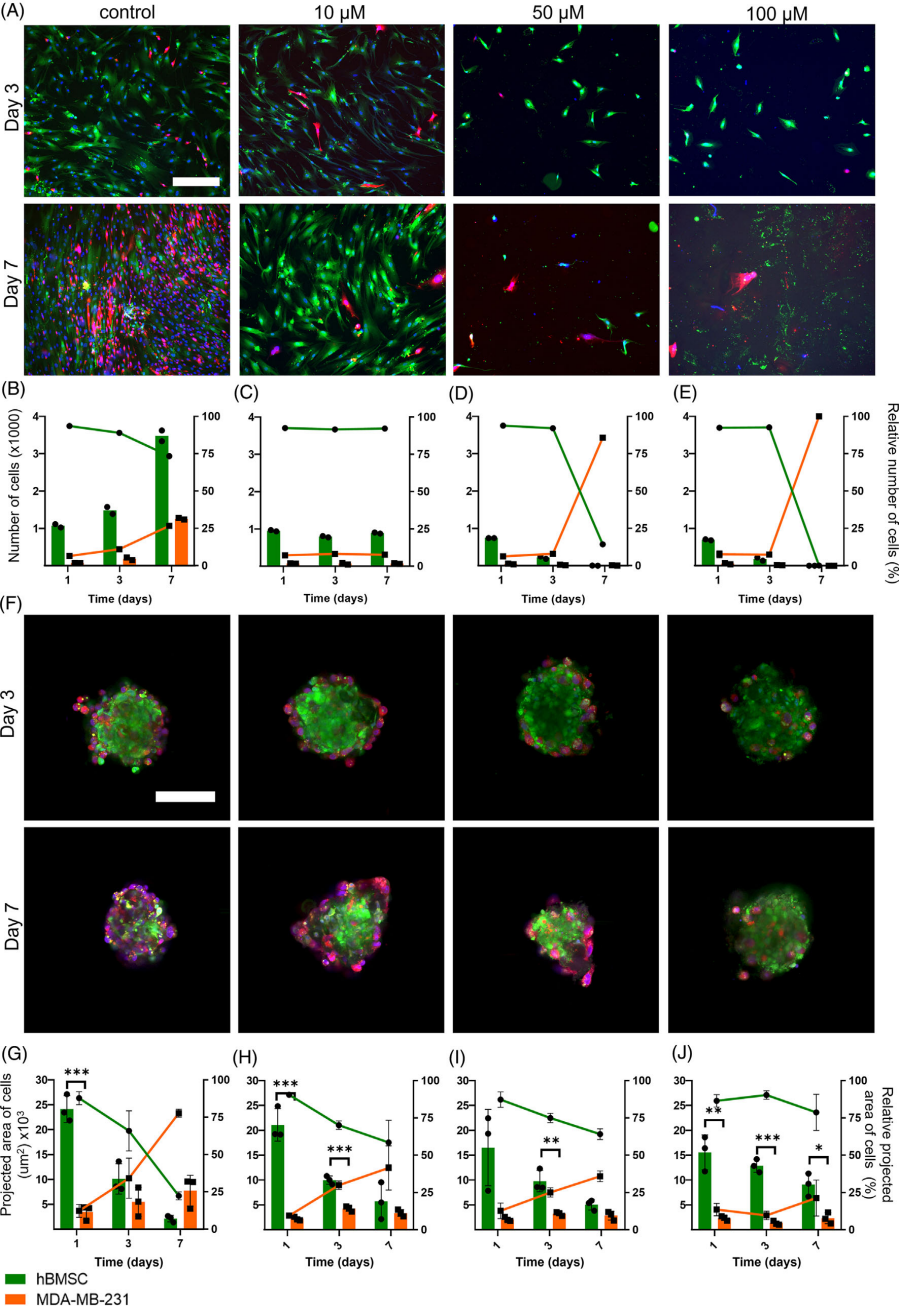
bBMSs show a lower susceptibility to cisplatin treatment (0, 10, 50 and 100 μM) compared to planar co‐cultures with breast cancer cells. Fluorescence images show treated planar cultures with breast cancer cells (A) while subsequent quantification of (relative) cell numbers (B–E) show that 10 μM of cisplatin was therapeutically effective. Fluorescence images show treated bBMSs (F) while subsequent quantification of (relative) cell areas (G–J) show that 50 and 100 μM of cisplatin were more therapeutically effective. Quantitative data are based on spheroids (*n* = 3) and wells (*n* = 2) for planar cultures and spheroids were statistically analysed using an independent *t*‐test on each timepoint; *p < 0.05; **p < 0.01; ***p < 0.001. Scale bar represents 300 μm in fluorescence images of planar cultures, and 100 μm in fluorescence images of spheroids. Cell numbers (planar) and cell areas (spheroids) are represented as bars and use the left y‐axis, while relative cell numbers (planar) and cell areas (spheroids) are represented as lines and use the right y‐axis. bBMS, breast BMS; BMS, bone metastasis spheroid.

**FIGURE 5:**
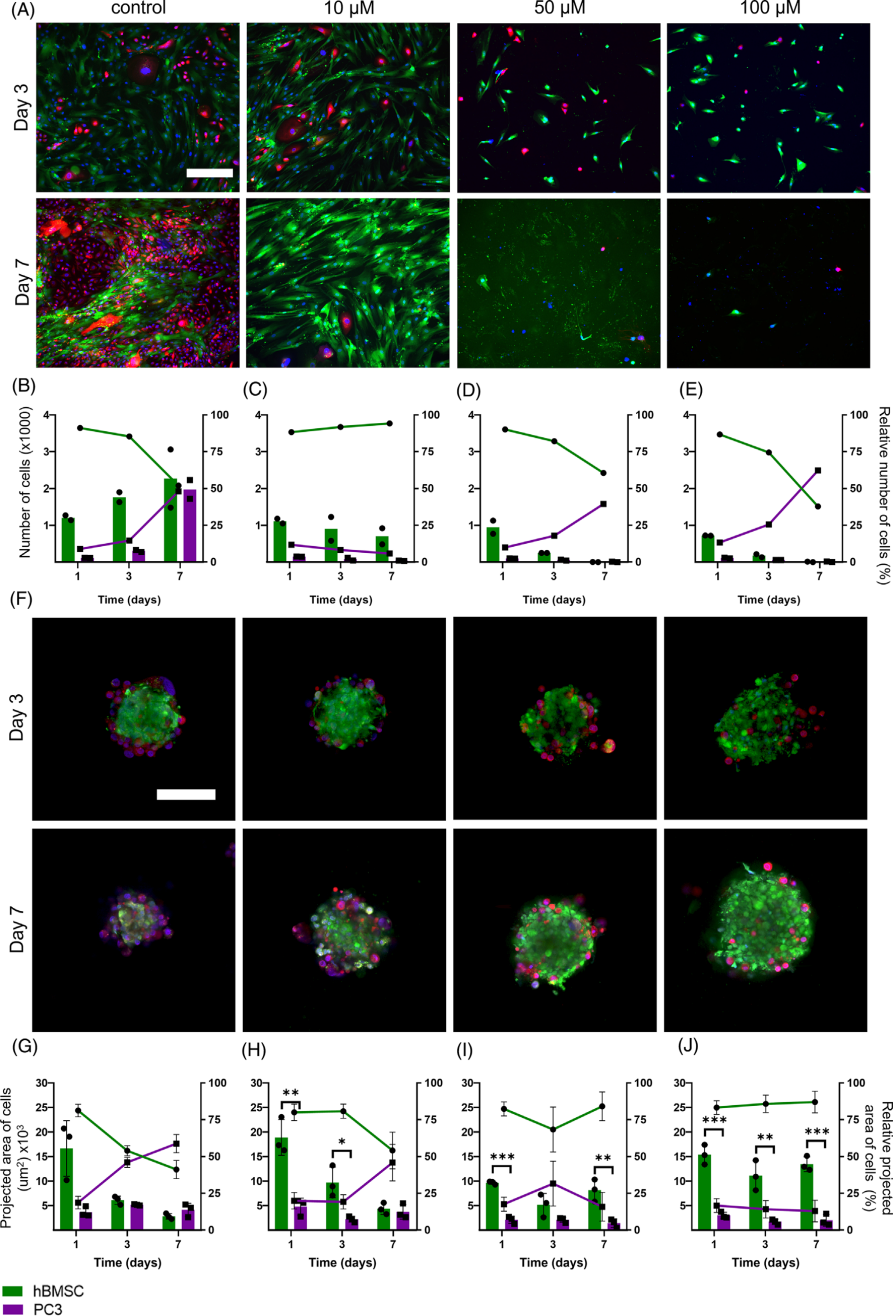
pBMSs show a lower susceptibility to cisplatin treatment (0, 10, 50 and 100 μM) compared to planar co‐cultures with prostate cancer cells. Fluorescence images show treated planar cultures with prostate cancer cells (A) while subsequent quantification of (relative) cell numbers (B–E) show that 10 μM of cisplatin was therapeutically effective. Fluorescence images show treated pBMSs (F) while subsequent quantification of (relative) cell areas (G–J) show that 50 and 100 μM of cisplatin were more therapeutically effective. Quantitative data are based on spheroids (*n* = 3) and wells (*n* = 2) for planar cultures and spheroids were statistically analysed using an independent *t*‐test on each timepoint; *p < 0.05; **p < 0.01; ***p < 0.001. Scale bar represents 300 μm in fluorescence images of planar cultures, and 100 μm in fluorescence images of spheroids. Cell numbers (planar) and cell areas (spheroids) are represented as bars and use the left y‐axis, while relative cell numbers (planar) and cell areas (spheroids) are represented as lines and use the right y‐axis. BMS, bone metastasis spheroid; pBMS, prostate cancer BMS.

To assess the response of our novel BMSs to chemotherapeutic drugs, we subjected BMSs and planar co‐cultures to increasing concentrations of cisplatin. Cisplatin is a chemotherapeutic drug commonly used for treatment of bone cancer^9^, ^101^ as well as for triple negative breast cancer^102^ and androgen receptor negative prostate cancer,^103^ where patients mostly receive an infusion for 24 h every 3 weeks.^13^ Furthermore, new therapies for bone metastases often include cisplatin (or similar platinum‐based compounds).^104^, ^105^, ^106^, ^107^ Cisplatin concentrations of 10, 25, 50 and 100 μM were supplemented to the culture medium for a 24 h exposure period, after which medium was refreshed and cells were cultured without cisplatin up to day 7. These concentrations and the exposure period were based on clinical usage of cisplatin (typically 100–120 mg/m^2^ per injection every two/three weeks^13^) and previous in vitro work on 2D and 3D cultures.^28^, ^58^, ^108^, ^109^, ^110^, ^111^, ^112^, ^113^, ^114^, ^115^, ^116^, ^117^, ^118^ On days 1, 3 and 7, fluorescence images were acquired to determine the presence of hBMSCs and cancer cells over time (Figures 4A,F and 5A,F). These fluorescence images were quantified resulting in measured cellular numbers and areas and their relative cellular quantities (Figures 4B–E,G–J and 5B–E,G–J).

Planar co‐cultures with either breast or prostate cancer cells showed that cisplatin effectively eliminated cancer cells at a concentration of 10 μM, without affecting hBMSCs, whereas higher concentrations of cisplatin (>50 μM) were required to achieve a similar effect in BMSs. Planar co‐cultures with 10 μM of cisplatin treatment showed ~7% of cancer cells at all timepoints, while 50 and 100 μM of cisplatin treatment caused considerable cell death and subsequent loss of both the hBMSCs and cancer cells. Interestingly, BMSs showed a different response to cisplatin treatment. Firstly, the response was delayed compared to the planar co‐cultures as no obvious differences among the various cisplatin concentrations were observed at day 3, but cisplatin efficacy was apparent at day 7. Cancer cells in BMSs treated with 10 μM of cisplatin kept proliferating similar to controls, showing higher relative cancer cell quantities compared to planar co‐cultures treated with this cisplatin concentration. This observation was in contrast to treatment with 100 μM cisplatin, which decreased cancer cell quantities in BMSs to only 21% and 13% for breast and prostate cancer cells at day 7, respectively (no significant differences found between relative cancer cell quantities over time for both bBMSs and pBMSs). Cell viability of BMSs after cisplatin treatment was further assessed using ATP measurements (Figure S6) on days 1, 3 and 7, and compared to control cultures without cisplatin treatment. Treatment with 100 μM of cisplatin resulted in 13% and 9% relative ATP content for bBMSs and pBMSs, respectively. These low ATP values are likely due to cisplatin effects on both cancer cells and hBMSC. However, as the proliferative cancer cells likely contributed to a larger extent to the total ATP content of the BMSs compared to the more quiescent hBMSCs,^34^, ^35^, ^73^ the decrease of ATP in cisplatin‐treated BMSs could be interpreted as a loss of mostly cancer cells.

**FIGURE 6:**
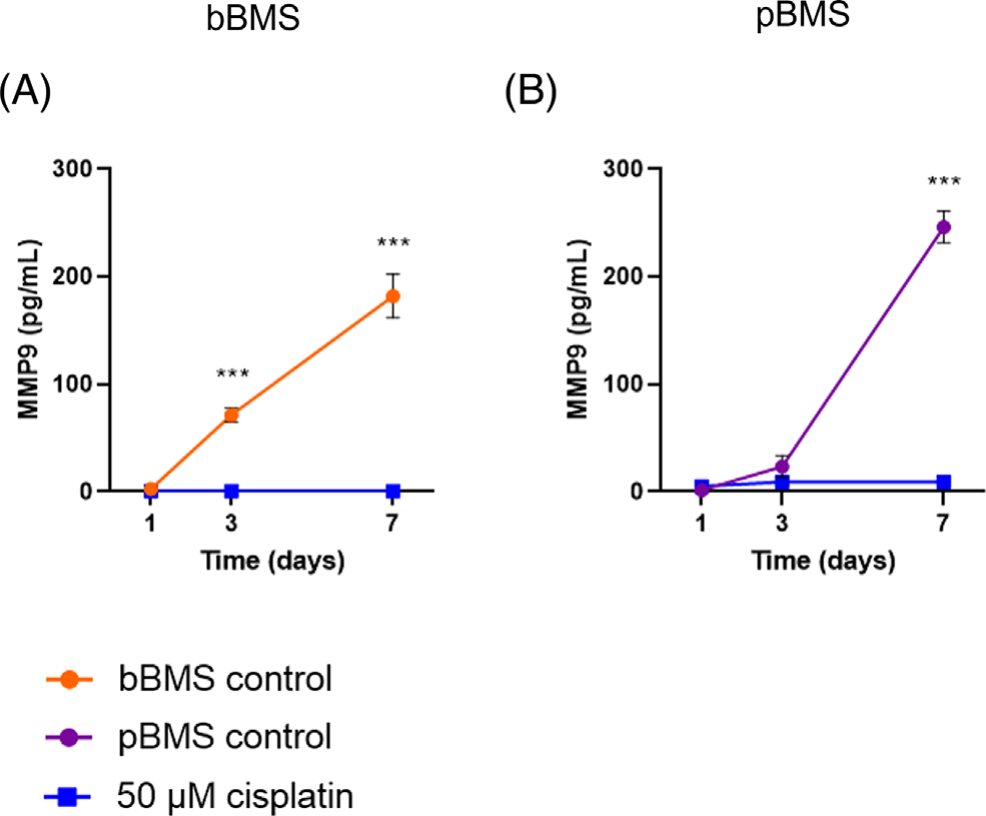
MMP9 measured in supernatant of bBMS (A) and pBMS (B) show a massive cumulative increase over time for the control conditions, while BMS treated with cisplatin (50 μM) show low to no MMP9 expression. Quantitative data based on (*n* = 3) spheroids and were statistically analysed using an independent *t*‐test on each timepoint; ***p < 0.001. bBMS, breast BMS; BMS, bone metastasis spheroid.

Furthermore, we measured MMP‐9 in the supernatant of both the bBMS and pBMS (Figure 6A,B) treated with cisplatin (0 and 50 μM). MMP9 is a biomarker for EMT signalling as well as for progression of bone metastasis.^51^, ^53^, ^54^, ^119^, ^120^, ^121^, ^122^ We found a massive increase, namely up to a cumulative amount of ~180 and ~ 250 pg/mL for bBMS and pBMS, respectively, on day 7. However, when treated with cisplatin the measured MMP‐9 remained close to 0 for the whole culture period. These findings corroborates our measured cancer cell proliferation (Figure 2), and the subsequent cancer cell suppression when treated with cisplatin (Figures 4 and 5) using the confocal microscopy techniques. Nevertheless, a direct relation to MMP‐9 expression to cancer cell number cannot be made, as cancer cells themselves can also increase their MMP‐9 production.

Lower susceptibility to chemotherapy in 3D cultures compared to 2D cultures is in line with previous reports for both breast^22^, ^24^, ^26^, ^27^, ^28^ and prostate^23^, ^25^ cancer cells. However, this lower susceptibility to chemotherapeutics was mostly related to a reduced proliferation of the cancer cells upon 3D culture, which is in contrast to the cancer cell proliferation observed in the BMSs and other 3D co‐cultures containing hBMSCs.^51^, ^88^ Other factors of the BMS could contribute to the lower susceptibility of cancer cells to chemotherapeutics, such as the exposure of the cells to the cisplatin. In a planar culture, all cells are evenly exposed to the culture medium containing cisplatin, while in BMSs peripheral cells are most likely exposed more efficiently to cisplatin compared to cells in the interior of BMSs. Furthermore, as shown previously,^46^, ^96^ the inclusion of healthy cell types in other 3D cultures of cancer cells, caused lower cancer cell susceptibilities to various chemotherapeutics in other models as well. Thus, besides supporting cancer cell proliferation, the co‐culture and three dimensionality of BMSs also altered the cancer cell response to cisplatin treatment. This lower susceptibility of cancer cells in the BMSs to chemotherapeutics, where only dosages of 50 and 100 μM of cisplatin kill cancer cells effectively, is clinically more relevant than the response of cancer cells and healthy cells in planar co‐cultures at these concentrations, where both cell types were completely eliminated.

Compared to other types of bone metastasis models used for screening of novel therapies, BMSs are easy to create, maintain, quantitatively characterise and image. Other models either require relatively long culture times (e.g., 8–12 weeks) to create human osteoblast‐derived microtissues^123^ or multiple biomaterials and processing steps.^43^

## 4 CONCLUSION AND OUTLOOK

Combinations of hBMSCs and breast or prostate cancer cells showed facile formation of stable BMSs within 24 h. Breast cancer metastatic spheroids decreased in size and remained largely circular up to 7 days in culture, while prostate cancer metastatic spheroids slightly increased in size and gradually lost their circularity. For both breast cancer and prostate cancer BMSs, cancer cells showed higher cell proliferation rates compared to their planar co‐culture counterparts. Despite the higher cellular proliferation within the BMSs, cancer cells were less susceptible to cisplatin treatment, while planar co‐cultures showed effective cancer cell elimination already at low cisplatin concentrations. Based on our results, it can be concluded that BMSs hold strong promise as 3D in vitro model for screening new therapies to treat bone metastases and personalised medicine by implementing cells from patient biopsies.

## AUTHOR CONTRIBUTIONS

**Ceri‐Anne E Suurmond**: Conceptualisation (equal); data curation (equal); formal analysis (equal); methodology (equal); visualisation (equal); writing—original draft (lead); writing—review & editing (equal). **Sander CG Leeuwenburgh**: Conceptualisation (equal); funding acquisition (lead); methodology (supporting); project administration (lead); supervision (equal); writing—review & editing (equal). **Jeroen JJP van den Beucken**: Conceptualisation (lead); methodology (lead); project administration (supporting); supervision (lead); writing—review & editing (equal).

## FUNDING INFORMATION

This publication is part of the project Colloidal self‐healing composites: towards a new class of biomaterials for regeneration of diseased bone (ColBioBone; project number 17835) of the research programme NWO Talent Programme Vici AES 2019, which is financed by the Dutch Research Council (NWO).

## CONFLICT OF INTEREST STATEMENT

All authors declare that they have no conflicts of interest.

## Supporting information


**DATA S1:** Supporting Information.

## Data Availability

The data that support the findings of this study are available from the corresponding author upon reasonable request.

## References

[1] Coleman RE , Croucher PI , Padhani AR , et al. Bone metastases. Nat Rev Dis Primers. 2020;6(1):83. 33060614 10.1038/s41572-020-00216-3

[2] Tsukamoto S , Kido A , Tanaka Y , et al. Current overview of treatment for metastatic bone disease. Curr Oncol. 2021;28(5):3347‐3372. 34590591 10.3390/curroncol28050290 PMC8482272

[3] Svensson E , Christiansen CF , Ulrichsen SP , Rørth MR , Sørensen HT . Survival after bone metastasis by primary cancer type: a Danish population‐based cohort study. BMJ Open. 2017;7(9):e016022. 10.1136/bmjopen-2017-016022 PMC5595184 28893744

[4] Castaneda M , den Hollander P , Kuburich NA , Rosen JM , Mani SA . Mechanisms of cancer metastasis. Semin Cancer Biol. 2022;87:17‐31. 36354098 10.1016/j.semcancer.2022.10.006

[5] Esposito M , Guise T , Kang Y . The biology of bone metastasis. Cold Spring Harb Perspect Med. 2018;8(6):A031252. 10.1101/cshperspect.a031252 PMC5980796 29101110

[6] Roodman GD . Mechanisms of bone metastasis. N Engl J Med. 2004;350(16):1655‐1664. 15084698 10.1056/NEJMra030831

[7] Wang W , Yang X , Dai J , Lu Y , Zhang J , Keller ET . Prostate cancer promotes a vicious cycle of bone metastasis progression through inducing osteocytes to secrete GDF15 that stimulates prostate cancer growth and invasion. Oncogene. 2019;38(23):4540‐4559. 30755731 10.1038/s41388-019-0736-3 PMC9097780

[8] Soeharno H , Povegliano L , Choong PF . Multimodal treatment of bone metastasis—a surgical perspective. Front Endocrinol (Lausanne). 2018;9:518. 30245668 10.3389/fendo.2018.00518 PMC6137681

[9] Bădilă AE , Rădulescu DM , Niculescu A‐G , Grumezescu AM , Rădulescu M , Rădulescu AR . Recent advances in the treatment of bone metastases and primary bone tumors: an up‐to‐date review. Cancer. 2021;13(16):4229. 10.3390/cancers13164229 PMC8392383 34439383

[10] Krishnan CK , Kim H‐S , Yun JY , Cho HS , Park JW , Han I . Factors associated with local recurrence after surgery for bone metastasis to the extremities. J Surg Oncol. 2018;117(4):797‐804. 29044578 10.1002/jso.24880

[11] Zhu H , Luo H , Zhang W , Shen Z , Hu X , Zhu X . Molecular mechanisms of cisplatin resistance in cervical cancer. Drug Des Devel Ther. 2016;10:1885‐1895. 10.2147/DDDT.S106412 PMC4907638 27354763

[12] Agnello L , Tortorella S , d'Argenio A , et al. Optimizing cisplatin delivery to triple‐negative breast cancer through novel EGFR aptamer‐conjugated polymeric nanovectors. J Exp Clin Cancer Res. 2021;40(1):239. 34294133 10.1186/s13046-021-02039-w PMC8299618

[13] Zhang B , Zhang Y , Li R , Li J , Lu X , Zhang Y . The efficacy and safety comparison of first‐line chemotherapeutic agents (high‐dose methotrexate, doxorubicin, cisplatin, and ifosfamide) for osteosarcoma: a network meta‐analysis. J Orthop Surg Res. 2020;15(1):51. 32054494 10.1186/s13018-020-1576-0 PMC7020590

[14] Miyazaki J , Kawai K , Hayashi H , et al. The limited efficacy of methotrexate, actinomycin D and cisplatin (MAP) for patients with advanced testicular cancer. Jpn J Clin Oncol. 2003;33(8):391‐395. 14523058 10.1093/jjco/hyg074

[15] Meng J , Gu QP , Meng QF , et al. Efficacy of nimotuzumab combined with docetaxel‐cisplatin‐fluorouracil regimen in treatment of advanced oral carcinoma. Cell Biochem Biophys. 2014;68(1):181‐184. 23733674 10.1007/s12013-013-9686-5

[16] Sajjad H , Imtiaz S , Noor T , Siddiqui YH , Sajjad A , Zia M . Cancer models in preclinical research: a chronicle review of advancement in effective cancer research. Animal Model Exp Med. 2021;4(2):87‐103. 34179717 10.1002/ame2.12165 PMC8212826

[17] Gilazieva Z , Ponomarev A , Rutland C , Rizvanov A , Solovyeva V . Promising applications of tumor spheroids and organoids for personalized medicine. Cancers (Basel). 2020;12(10):2727. doi:10.3390/cancers12102727 PMC7598156 32977530

[18] Walmsley GG , Ransom RC , Zielins ER , et al. Stem cells in bone regeneration. Stem Cell Rev Rep. 2016;12(5):524‐529. 27250635 10.1007/s12015-016-9665-5 PMC5053855

[19] Pittenger MF , Discher DE , Péault BM , Phinney DG , Hare JM , Caplan AI . Mesenchymal stem cell perspective: cell biology to clinical progress. Npj. Regen Med. 2019;4(1):22. 10.1038/s41536-019-0083-6 PMC6889290 31815001

[20] Senthebane DA , Rowe A , Thomford NE , et al. The role of tumor microenvironment in chemoresistance: to survive, keep your enemies closer. Int J Mol Sci. 2017;18(7):1586. 28754000 10.3390/ijms18071586 PMC5536073

[21] Vis MAM , Ito K , Hofmann S . Impact of culture medium on cellular interactions in in vitro co‐culture systems. Front Bioeng Biotechnol. 2020;8:911. 32850750 10.3389/fbioe.2020.00911 PMC7417654

[22] Muguruma M , Teraoka S , Miyahara K , et al. Differences in drug sensitivity between two‐dimensional and three‐dimensional culture systems in triple‐negative breast cancer cell lines. Biochem Biophys Res Commun. 2020;533(3):268‐274. 32958246 10.1016/j.bbrc.2020.08.075

[23] Grayson KA , Jyotsana N , Ortiz‐Otero N , King MR . Overcoming TRAIL‐resistance by sensitizing prostate cancer 3D spheroids with taxanes. PLoS One. 2021;16(3):e0246733. 33661931 10.1371/journal.pone.0246733 PMC7932526

[24] Huang Z , Yu P , Tang J . Characterization of triple‐negative breast cancer MDA‐MB‐231 cell spheroid model. Onco Targets Ther. 2020;13:5395‐5405. 32606757 10.2147/OTT.S249756 PMC7295545

[25] Chambers KF , Mosaad EMO , Russell PJ , Clements JA , Doran MR . 3D cultures of prostate cancer cells cultured in a novel high‐throughput culture platform are more resistant to chemotherapeutics compared to cells cultured in monolayer. PLoS One. 2014;9(11):e111029. 25380249 10.1371/journal.pone.0111029 PMC4224379

[26] Breslin S , O'Driscoll L . The relevance of using 3D cell cultures, in addition to 2D monolayer cultures, when evaluating breast cancer drug sensitivity and resistance. Oncotarget. 2016;7(29):45745‐45756. 27304190 10.18632/oncotarget.9935 PMC5216757

[27] Lovitt CJ , Shelper TB , Avery VM . Evaluation of chemotherapeutics in a three‐dimensional breast cancer model. J Cancer Res Clin Oncol. 2015;141(5):951‐959. 25773123 10.1007/s00432-015-1950-1 PMC11823512

[28] Gomes LR , Rocha CRR , Martins DJ , et al. ATR mediates cisplatin resistance in 3D‐cultured breast cancer cells via translesion DNA synthesis modulation. Cell Death Dis. 2019;10(6):459. 31189884 10.1038/s41419-019-1689-8 PMC6561919

[29] Azimi T , Loizidou M , Dwek MV . Cancer cells grown in 3D under fluid flow exhibit an aggressive phenotype and reduced responsiveness to the anti‐cancer treatment doxorubicin. Sci Rep. 2020;10(1):12020. 32694700 10.1038/s41598-020-68999-9 PMC7374750

[30] Brodeur MN , Simeone K , Leclerc‐Deslauniers K , et al. Carboplatin response in preclinical models for ovarian cancer: comparison of 2D monolayers, spheroids, ex vivo tumors and in vivo models. Sci Rep. 2021;11(1):18183. 34521878 10.1038/s41598-021-97434-w PMC8440566

[31] Zanoni M , Cortesi M , Zamagni A , Arienti C , Pignatta S , Tesei A . Modeling neoplastic disease with spheroids and organoids. J Hematol Oncol. 2020;13(1):97. 32677979 10.1186/s13045-020-00931-0 PMC7364537

[32] Hsiao AY , Torisawa YS , Tung YC , et al. Microfluidic system for formation of PC‐3 prostate cancer co‐culture spheroids. Biomaterials. 2009;30(16):3020‐3027. 19304321 10.1016/j.biomaterials.2009.02.047 PMC2675053

[33] Yan X‐Z , van den Beucken J , Yuan C , Jansen J , Yang F . Spheroid formation and stemness preservation of human periodontal ligament cells on chitosan films. Oral Dis. 2018;24(6):1083‐1092. 29514415 10.1111/odi.12855

[34] Rybkowska P , Radoszkiewicz K , Kawalec M , et al. The metabolic changes between monolayer (2D) and three‐dimensional (3D) culture conditions in human mesenchymal stem/stromal cells derived from adipose tissue. Cells. 2023;12(1):178. doi:10.3390/cells12010178 PMC9818744 36611971

[35] Lewis NS , Lewis EE , Mullin M , Wheadon H , Dalby MJ , Berry CC . Magnetically levitated mesenchymal stem cell spheroids cultured with a collagen gel maintain phenotype and quiescence. J Tissue Eng. 2017;8:2041731417704428. 28616152 10.1177/2041731417704428 PMC5460809

[36] Fois MG , Tahmasebi Birgani ZN , Guttenplan APM , et al. Assessment of cell–material interactions in three dimensions through dispersed coaggregation of microsized biomaterials into tissue spheroids. Small. 2022;18(29):2202112. 10.1002/smll.202202112 35754160

[37] Sethakorn N , Heninger E , Sánchez‐de‐Diego C , et al. Advancing treatment of bone metastases through novel translational approaches targeting the bone microenvironment. Cancers (Basel). 2022;14(3):757. 10.3390/cancers14030757 PMC8833657 35159026

[38] Marturano‐Kruik A , Nava MM , Yeager K , et al. Human bone perivascular niche‐on‐a‐chip for studying metastatic colonization. Proc Natl Acad Sci USA. 2018;115(6):1256‐1261. 29363599 10.1073/pnas.1714282115 PMC5819403

[39] Hao S , Ha L , Cheng G , et al. A spontaneous 3D bone‐on‐a‐chip for bone metastasis study of breast cancer cells. Small. 2018;14(12):e1702787. 29399951 10.1002/smll.201702787

[40] Bersini S , Jeon JS , Dubini G , et al. A microfluidic 3D in vitro model for specificity of breast cancer metastasis to bone. Biomaterials. 2014;35(8):2454‐2461. 24388382 10.1016/j.biomaterials.2013.11.050 PMC3905838

[41] Jeon JS , Bersini S , Gilardi M , et al. Human 3D vascularized organotypic microfluidic assays to study breast cancer cell extravasation. Proc Natl Acad Sci USA. 2015;112(1):214‐219. 25524628 10.1073/pnas.1417115112 PMC4291627

[42] Choudhary S , Ramasundaram P , Dziopa E , et al. Human ex vivo 3D bone model recapitulates osteocyte response to metastatic prostate cancer. Sci Rep. 2018;8(1):17975. 30568232 10.1038/s41598-018-36424-x PMC6299475

[43] Mohseni Garakani M , Cooke ME , Weber MH , Wertheimer MR , Ajji A , Rosenzweig DH . A 3D, compartmental tumor‐stromal microenvironment model of patient‐derived bone metastasis. Int J Mol Sci. 2023;24(1):160. 10.3390/ijms24010160 PMC9820116 36613604

[44] Cui H , Esworthy T , Zhou X , et al. Engineering a novel 3D printed vascularized tissue model for investigating breast cancer metastasis to bone. Adv Healthc Mater. 2020;9(15):1900924. 10.1002/adhm.201900924 PMC7297662 31846231

[45] Angeloni V , Contessi N , De Marco C , et al. Polyurethane foam scaffold as in vitro model for breast cancer bone metastasis. Acta Biomater. 2017;63:306‐316. 28927931 10.1016/j.actbio.2017.09.017

[46] Paindelli C , Navone N , Logothetis CJ , Friedl P , Dondossola E . Engineered bone for probing organotypic growth and therapy response of prostate cancer tumoroids in vitro. Biomaterials. 2019;197:296‐304. 30682644 10.1016/j.biomaterials.2019.01.027 PMC7094882

[47] Zhu W , Wang M , Fu Y , Castro NJ , Fu SW , Zhang LG . Engineering a biomimetic three‐dimensional nanostructured bone model for breast cancer bone metastasis study. Acta Biomater. 2015;14:164‐174. 25528534 10.1016/j.actbio.2014.12.008

[48] Zhou X , Zhu W , Nowicki M , et al. 3D bioprinting a cell‐laden bone matrix for breast cancer metastasis study. ACS Appl Mater Interfaces. 2016;8(44):30017‐30026. 27766838 10.1021/acsami.6b10673

[49] Pathi SP , Lin DD , Dorvee JR , Estroff LA , Fischbach C . Hydroxyapatite nanoparticle‐containing scaffolds for the study of breast cancer bone metastasis. Biomaterials. 2011;32(22):5112‐5122. 21507478 10.1016/j.biomaterials.2011.03.055 PMC3613283

[50] Marlow R , Honeth G , Lombardi S , et al. A novel model of dormancy for bone metastatic breast cancer cells. Cancer Res. 2013;73(23):6886‐6899. 24145351 10.1158/0008-5472.CAN-13-0991

[51] Fitzgerald KA , Guo J , Raftery RM , et al. Nanoparticle‐mediated siRNA delivery assessed in a 3D co‐culture model simulating prostate cancer bone metastasis. Int J Pharm. 2016;511(2):1058‐1069. 27492023 10.1016/j.ijpharm.2016.07.079

[52] Kar S , Molla MS , Katti DR , Katti KS . Tissue‐engineered nanoclay‐based 3D in vitro breast cancer model for studying breast cancer metastasis to bone. J Tissue Eng Regen Med. 2019;13(2):119‐130. 30466156 10.1002/term.2773

[53] Taubenberger AV , Quent VM , Thibaudeau L , Clements JA , Hutmacher DW . Delineating breast cancer cell interactions with engineered bone microenvironments. J Bone Miner Res. 2013;28(6):1399‐1411. 23362043 10.1002/jbmr.1875

[54] Reichert JC , Quent VM , Burke LJ , Stansfield SH , Clements JA , Hutmacher DW . Mineralized human primary osteoblast matrices as a model system to analyse interactions of prostate cancer cells with the bone microenvironment. Biomaterials. 2010;31(31):7928‐7936. 20688384 10.1016/j.biomaterials.2010.06.055

[55] Lescarbeau RM , Seib FP , Prewitz M , Werner C , Kaplan DL . In vitro model of metastasis to bone marrow mediates prostate cancer castration resistant growth through paracrine and extracellular matrix factors. PLoS One. 2012;7(8):e40372. 22870197 10.1371/journal.pone.0040372 PMC3411611

[56] Qiao H , Tang T . Engineering 3D approaches to model the dynamic microenvironments of cancer bone metastasis. Bone Res. 2018;6(1):3. 29507817 10.1038/s41413-018-0008-9 PMC5826951

[57] Decarli MC , Amaral R , Santos DPD , et al. Cell spheroids as a versatile research platform: formation mechanisms, high throughput production, characterization and applications. Biofabrication. 2021;13(3):032002. doi:10.1088/1758-5090/abe6f2 33592595

[58] Antunes J , Gaspar VM , Ferreira L , et al. In‐air production of 3D co‐culture tumor spheroid hydrogels for expedited drug screening. Acta Biomater. 2019;94:392‐409. 31200118 10.1016/j.actbio.2019.06.012

[59] Tai S , Sun Y , Squires JM , et al. PC3 is a cell line characteristic of prostatic small cell carcinoma. Prostate. 2011;71(15):1668‐1679. 21432867 10.1002/pros.21383 PMC3426349

[60] Park SH , Eber MR , Shiozawa Y . Models of prostate cancer bone metastasis. Methods Mol Biol. 2019;1914:295‐308. 30729472 10.1007/978-1-4939-8997-3_16 PMC6738334

[61] DeCastro AJL , Pranda MA , Gray KM , et al. Morphological phenotyping of organotropic brain‐ and bone‐seeking triple negative metastatic breast tumor cells. Front Cell Dev Biol. 2022;10:790410. 35252171 10.3389/fcell.2022.790410 PMC8891987

[62] Ma J , van den Beucken JJ , Both SK , et al. Osteogenic capacity of human BM‐MSCs, AT‐MSCs and their co‐cultures using HUVECs in FBS and PL supplemented media. J Tissue Eng Regen Med. 2015;9(7):779‐788. 23364774 10.1002/term.1704

[63] Dominici M , Le Blanc K , Mueller I , et al. Minimal criteria for defining multipotent mesenchymal stromal cells. The International Society for Cellular Therapy position statement. Cytotherapy. 2006;8(4):315‐317. 16923606 10.1080/14653240600855905

[64] Schindelin J , Arganda‐Carreras I , Frise E , et al. Fiji: an open‐source platform for biological‐image analysis. Nat Methods. 2012;9(7):676‐682. 22743772 10.1038/nmeth.2019 PMC3855844

[65] Mihara H , Kugawa M , Sayo K , et al. Improved oxygen supply to multicellular spheroids using a gas‐permeable plate and embedded hydrogel beads. Cells. 2019;8(6):525. 31159231 10.3390/cells8060525 PMC6627619

[66] Ollion J , Cochennec J , Loll F , Escudé C , Boudier T . TANGO: a generic tool for high‐throughput 3D image analysis for studying nuclear organization. Bioinformatics. 2013;29(14):1840‐1841. 23681123 10.1093/bioinformatics/btt276 PMC3702251

[67] Vindin H , Bischof L , Gunning P , Stehn J . Validation of an algorithm to quantify changes in Actin cytoskeletal organization. SLAS Discov. 2014;19(3):354‐368. 10.1177/1087057113503494 24019255

[68] Keller F , Rudolf R , Hafner M . Towards optimized breast cancer 3D spheroid mono‐ and co‐culture models for pharmacological research and screening. J Cell Biotechnol. 2019;5:89‐101.

[69] Raghavan S , Mehta P , Horst EN , Ward MR , Rowley KR , Mehta G . Comparative analysis of tumor spheroid generation techniques for differential in vitro drug toxicity. Oncotarget. 2016;7(13):16948‐16961. 26918944 10.18632/oncotarget.7659 PMC4941362

[70] Leung BM , Lesher‐Perez SC , Matsuoka T , Moraes C , Takayama S . Media additives to promote spheroid circularity and compactness in hanging drop platform. Biomater Sci. 2015;3(2):336‐344. 26218124 10.1039/c4bm00319e

[71] Kuo C‐T , Wang J‐Y , Lin Y‐F , Wo AM , Chen BPC , Lee H . Three‐dimensional spheroid culture targeting versatile tissue bioassays using a PDMS‐based hanging drop array. Sci Rep. 2017;7(1):4363. 28663555 10.1038/s41598-017-04718-1 PMC5491519

[72] Schmitz C , Potekhina E , Irianto T , Belousov VV , Lavrentieva A . Hypoxia onset in mesenchymal stem cell spheroids: monitoring with hypoxia reporter cells. Front Bioeng Biotechnol. 2021;9:611837. 10.3389/fbioe.2021.611837 PMC7892969 33614611

[73] Murphy KC , Hung BP , Browne‐Bourne S , et al. Measurement of oxygen tension within mesenchymal stem cell spheroids. J R Soc Interface. 2017;14(127):20160851. 28179546 10.1098/rsif.2016.0851 PMC5332570

[74] Singh SK , Abbas S , Saxena AK , Tiwari S , Sharma LK , Tiwari M . Critical role of three‐dimensional tumorsphere size on experimental outcome. Biotechniques. 2020;69(5):333‐338. 33000639 10.2144/btn-2020-0081

[75] Mukomoto R , Nashimoto Y , Terai T , et al. Oxygen consumption rate of tumour spheroids during necrotic‐like core formation. Analyst. 2020;145(19):6342‐6348. 32716439 10.1039/d0an00979b

[76] Costard LS , Hosn RR , Ramanayake H , O'Brien FJ , Curtin CM . Influences of the 3D microenvironment on cancer cell behaviour and treatment responsiveness: a recent update on lung, breast and prostate cancer models. Acta Biomater. 2021;132:360‐378. doi:10.1016/j.actbio.2021.01.023 33484910

[77] Laranga R , Duchi S , Ibrahim T , Guerrieri AN , Donati DM , Lucarelli E . Trends in bone metastasis modeling. Cancers (Basel). 2020;12(8):2315. doi:10.3390/cancers12082315 PMC7464021 32824479

[78] Poornima K , Francis AP , Hoda M , et al. Implications of three‐dimensional cell culture in cancer therapeutic research. Front Oncol. 2022;12:891673. 10.3389/fonc.2022.891673 PMC9133474 35646714

[79] Tsai AC , Liu Y , Yuan X , Ma T . Compaction, fusion, and functional activation of three‐dimensional human mesenchymal stem cell aggregate. Tissue Eng Part A. 2015;21(9‐10):1705‐1719. 25661745 10.1089/ten.tea.2014.0314 PMC4426301

[80] Saraiva DP , Matias AT , Braga S , Jacinto A , Cabral MG . Establishment of a 3D Co‐culture with MDA‐MB‐231 breast cancer cell line and patient‐derived immune cells for application in the development of immunotherapies. Front Oncol. 2020;10:1543. 32974189 10.3389/fonc.2020.01543 PMC7482668

[81] Cavaco M , Fraga P , Valle J , Andreu D , Castanho M , Neves V . Development of breast cancer spheroids to evaluate cytotoxic response to an anticancer peptide. Pharmaceutics. 2021;13(11):1863. doi:10.3390/pharmaceutics13111863 PMC8619419 34834277

[82] Dang PN , Dwivedi N , Yu X , et al. Guiding chondrogenesis and osteogenesis with mineral‐coated hydroxyapatite and BMP‐2 incorporated within high‐density hMSC aggregates for bone regeneration. ACS Biomater Sci Eng. 2016;2(1):30‐42. 33418642 10.1021/acsbiomaterials.5b00277

[83] Taqi SA , Sami SA , Sami LB , Zaki SA . A review of artifacts in histopathology. J Oral Maxillofac Pathol. 2018;22(2):279. 10.4103/jomfp.JOMFP_125_15 PMC6097380 30158787

[84] Mandel K , Yang Y , Schambach A , Glage S , Otte A , Hass R . Mesenchymal stem cells directly interact with breast cancer cells and promote tumor cell growth in vitro and in vivo. Stem Cells Dev. 2013;22(23):3114‐3127. 23895436 10.1089/scd.2013.0249

[85] Li Q , Rycaj K , Chen X , Tang DG . Cancer stem cells and cell size: a causal link? Semin Cancer Biol. 2015;35:191‐199. 26241348 10.1016/j.semcancer.2015.07.002 PMC4651715

[86] Doğan A , Demirci S , Apdik H , Apdik EA , Şahin F . Mesenchymal stem cell isolation from pulp tissue and co‐culture with cancer cells to study their interactions. J Vis Exp. 2019;7(143):e58825. doi:doi:10.3791/58825 30663693

[87] Chong MS , Lim J , Goh J , Sia MW , Chan JK , Teoh SH . Cocultures of mesenchymal stem cells and endothelial cells as organotypic models of prostate cancer metastasis. Mol Pharm. 2014;11(7):2126‐2133. 24779855 10.1021/mp500141b

[88] Bartosh TJ , Ullah M , Zeitouni S , Beaver J , Prockop DJ . Cancer cells enter dormancy after cannibalizing mesenchymal stem/stromal cells (MSCs). Proc Natl Acad Sci USA. 2016;113(42):E6447‐e6456. 27698134 10.1073/pnas.1612290113 PMC5081643

[89] Fais S , Overholtzer M . Cell‐in‐cell phenomena, cannibalism, and autophagy: is there a relationship? Cell Death Dis. 2018;9(2):95. 29367622 10.1038/s41419-017-0111-7 PMC5833709

[90] Sieh S , Taubenberger AV , Lehman ML , Clements JA , Nelson CC , Hutmacher DW . Paracrine interactions between LNCaP prostate cancer cells and bioengineered bone in 3D in vitro culture reflect molecular changes during bone metastasis. Bone. 2014;63:121‐131. 24530694 10.1016/j.bone.2014.02.001

[91] Aravindhan S , Ejam SS , Lafta MH , Markov A , Yumashev AV , Ahmadi M . Mesenchymal stem cells and cancer therapy: insights into targeting the tumour vasculature. Cancer Cell Int. 2021;21(1):158. 33685452 10.1186/s12935-021-01836-9 PMC7938588

[92] Lee HY , Hong IS . Double‐edged sword of mesenchymal stem cells: cancer‐promoting versus therapeutic potential. Cancer Sci. 2017;108(10):1939‐1946. 28756624 10.1111/cas.13334 PMC5623746

[93] Xuan X , Tian C , Zhao M , Sun Y , Huang C . Mesenchymal stem cells in cancer progression and anticancer therapeutic resistance. Cancer Cell Int. 2021;21(1):595. 34736460 10.1186/s12935-021-02300-4 PMC8570012

[94] Yoneda T , Hiasa M , Nagata Y , Okui T , White F . Contribution of acidic extracellular microenvironment of cancer‐colonized bone to bone pain. Biochim Biophys Acta. 2015;1848(10):2677‐2684. 25687976 10.1016/j.bbamem.2015.02.004 PMC5356024

[95] Avnet S , Di Pompo G , Lemma S , Baldini N . Cause and effect of microenvironmental acidosis on bone metastases. Cancer Metastasis Rev. 2019;38(1):133‐147. 30825056 10.1007/s10555-019-09790-9 PMC6647382

[96] Mosaad E , Chambers K , Futrega K , Clements J , Doran MR . Using high throughput microtissue culture to study the difference in prostate cancer cell behavior and drug response in 2D and 3D co‐cultures. BMC Cancer. 2018;18(1):592. 29793440 10.1186/s12885-018-4473-8 PMC5968610

[97] Duś‐Szachniewicz K , Gdesz‐Birula K , Rymkiewicz G . Development and characterization of 3D hybrid spheroids for the investigation of the crosstalk between B‐cell non‐hodgkin lymphomas and mesenchymal stromal cells. Onco Targets Ther. 2022;15:683‐697. 35747403 10.2147/OTT.S363994 PMC9213039

[98] Foty RA , Steinberg MS . The differential adhesion hypothesis: a direct evaluation. Dev Biol. 2005;278(1):255‐263. 15649477 10.1016/j.ydbio.2004.11.012

[99] Foty RA , Steinberg MS . Differential adhesion in model systems. Wiley Interdiscip Rev Dev Biol. 2013;2(5):631‐645. 24014451 10.1002/wdev.104

[100] Kaszak I , Witkowska‐Piłaszewicz O , Niewiadomska Z , Dworecka‐Kaszak B , Ngosa Toka F , Jurka P . Role of cadherins in cancer—a review. Int J Mol Sci. 2020;21(20):7624. doi:10.3390/ijms21207624 PMC7589192 33076339

[101] Smrke A , Anderson PM , Gulia A , Gennatas S , Huang PH , Jones RL . Future directions in the treatment of osteosarcoma. Cells. 2021;10(1):172. 33467756 10.3390/cells10010172 PMC7829872

[102] Poggio F , Bruzzone M , Ceppi M , et al. Platinum‐based neoadjuvant chemotherapy in triple‐negative breast cancer: a systematic review and meta‐analysis. Ann Oncol. 2018;29(7):1497‐1508. 29873695 10.1093/annonc/mdy127

[103] Chou F‐J , Lin C , Tian H , et al. Preclinical studies using cisplatin/carboplatin to restore the enzalutamide sensitivity via degrading the androgen receptor splicing variant 7 (ARv7) to further suppress enzalutamide resistant prostate cancer. Cell Death Dis. 2020;11(11):942. 33139720 10.1038/s41419-020-02970-4 PMC7606511

[104] Shao H , Varamini P . Breast cancer bone metastasis: a narrative review of emerging targeted drug delivery systems. Cells. 2022;11(3):388. 35159207 10.3390/cells11030388 PMC8833898

[105] Yu Y , Sun L , Tang Y , et al. Preparation of cisplatin delivery calcium phosphate nanoparticles using poly(Pt(IV) prodrug) as the payload. Mater Today Commun. 2022;33:104283.

[106] Zakeri N , Rezaie HR , Javadpour J , Kharaziha M . Cisplatin loaded polycaprolactone—zeolite nanocomposite scaffolds for bone cancer treatment. J Sci Adv Mater Devices. 2021;7:100377.

[107] Barbanente A , Gandin V , Ditaranto N , et al. A Pt(IV) prodrug of kiteplatin with the bone‐targeting pyrophosphate ligand. Inorg Chim Acta. 2019;494:98‐104.

[108] Wang S , Xie J , Li J , Liu F , Wu X , Wang Z . Cisplatin suppresses the growth and proliferation of breast and cervical cancer cell lines by inhibiting integrin β5‐mediated glycolysis. Am J Cancer Res. 2016;6(5):1108‐1117. 27294003 PMC4889724

[109] Baek N , Seo OW , Lee J , Hulme J , An SS . Real‐time monitoring of cisplatin cytotoxicity on three‐dimensional spheroid tumor cells. Drug Des Devel Ther. 2016;10:2155‐2165. 10.2147/DDDT.S108004 PMC4938242 27445462

[110] Barbanente A , Nadar RA , Esposti LD , et al. Platinum‐loaded, selenium‐doped hydroxyapatite nanoparticles selectively reduce proliferation of prostate and breast cancer cells co‐cultured in the presence of stem cells. J Mater Chem B. 2020;8(14):2792‐2804. 32159578 10.1039/d0tb00390e

[111] Nadar RA , Asokan N , Degli Esposti L , et al. Preclinical evaluation of platinum‐loaded hydroxyapatite nanoparticles in an embryonic zebrafish xenograft model. Nanoscale. 2020;12(25):13582‐13594. 32555916 10.1039/d0nr04064a

[112] Wang Z , Nogueira LP , Haugen HJ , et al. Dual‐functional porous and cisplatin‐loaded polymethylmethacrylate cement for reconstruction of load‐bearing bone defect kills bone tumor cells. Bioact Mater. 2022;15:120‐130. 35386344 10.1016/j.bioactmat.2021.12.023 PMC8941180

[113] Bhatavdekar O , Godet I , Gilkes D , Sofou S . The rate of cisplatin dosing affects the resistance and metastatic potential of triple negative breast cancer cells, independent of hypoxia. Pharmaceutics. 2022;14(10):2184. doi:10.3390/pharmaceutics14102184 PMC9611288 36297617

[114] Ata FK , Yalcin S . The cisplatin, 5‐fluorouracil, irinotecan, and gemcitabine treatment in resistant 2D and 3D model triple negative breast cancer cell line: ABCG2 expression data. Anti Cancer Agents Med Chem. 2022;22(2):371‐377. 10.2174/1871520621666210727105431 34315389

[115] Malhão F , Ramos AA , Macedo AC , Rocha E . Cytotoxicity of seaweed compounds, alone or combined to reference drugs, against breast cell lines cultured in 2D and 3D. Toxics. 2021;9(2):24. 10.3390/toxics9020024 PMC7912033 33572635

[116] Malhão F , Macedo AC , Costa C , Rocha E , Ramos AA . Fucoxanthin holds potential to become a drug adjuvant in breast cancer treatment: evidence from 2D and 3D cell cultures. Molecules [Internet]. 2021;26(14):4288. doi:10.3390/molecules2614428 PMC8304772 34299562

[117] Boccellino M , Ambrosio P , Ballini A , et al. The role of curcumin in prostate cancer cells and derived spheroids. Cancers (Basel). 2022;14(14):3348. doi:10.3390/cancers14143348 PMC9320241 35884410

[118] Gold JM , Raja A . Cisplatin. StatPearls. Treasure Island (FL): StatPearls Publishing Copyright © 2023. StatPearls Publishing LLC; 2023.

[119] Huang H . Matrix metalloproteinase‐9 (MMP‐9) as a cancer biomarker and MMP‐9 biosensors: recent advances. Sensors (Basel). 2018;18(10):32‐49. 10.3390/s18103249 PMC6211011 30262739

[120] Holen I , Nutter F , Wilkinson JM , Evans CA , Avgoustou P , Ottewell PD . Human breast cancer bone metastasis in vitro and in vivo: a novel 3D model system for studies of tumour cell‐bone cell interactions. Clin Exp Metastasis. 2015;32(7):689‐702. 26231669 10.1007/s10585-015-9737-y

[121] Sieh S , Lubik AA , Clements JA , Nelson CC , Hutmacher DW . Interactions between human osteoblasts and prostate cancer cells in a novel 3D in vitro model. Organogenesis. 2010;6(3):181‐188. 21197221 10.4161/org.6.3.12041 PMC2946051

[122] Yousef EM , Tahir MR , St‐Pierre Y , Gaboury LA . MMP‐9 expression varies according to molecular subtypes of breast cancer. BMC Cancer. 2014;14:609. 25151367 10.1186/1471-2407-14-609 PMC4150970

[123] Bock N , Kryza T , Shokoohmand A , et al. In vitro engineering of a bone metastases model allows for study of the effects of antiandrogen therapies in advanced prostate cancer. Sci Adv. 2021;7(27):eabg2564. 10.1126/sciadv.abg2564 PMC8245033 34193425

