# Supplementary material for: Modelling bone metastasis in spheroids to study cancer progression and screen cisplatin efficacy
Source: Cell Prolif. 2024 Jun 20;57(9):e13693. doi: 10.1111/cpr.13693 (PMC11503253; doi:10.1111/cpr.13693)
Supplement: Supplementary file 1 — DATA S1: Supporting Information. [file CPR-57-e13693-s001.pdf]

Supplementary files

1. Fiji protocols

1.1. Morphology analysis of BMS

1. Set scale (analyze → set scale…) of 470,5307 pixels per 1000 µm and tick ‘Global’.
2. Set measurements (analyze → set measurements…) by selecting the following: area, shape descriptors, perimeter, feret’s diameter, and add to overlay.
3. Make sure black background at options for binary is not ticked (process → binary → options…)
4. Make binary images of the spheroids with batch processing (process → batch →macro), select a new folder and run the following script:

```
run("Minimum...", "radius=1");
run("8-bit");
run("Auto Threshold", "method=Huang white");
run("Fill Holes");
run("Close-");
run("Fill Holes");
```

5. Exclude the images where the background is merged with the BMS. Don’t use watershed function as this can remove parts of the spheroids, especially in earlier timepoints.
6. Open the new binary images in FIJI, and analyze the spheroids morphology by analyzing particles (analyze → analyze particles…), and put in the following as settings:
    a. Size: 1000-150000
    b. Circularity: 0.00-1.00
    c. Show: overlay
    d. Tick the following: display results, clear results, exclude on edges, and include holes.

1.2. Cellular quantities and ratios in planar co-cultures

1. Make sure black background at options for binary is ticked (process → binary → options…)
2. Open the images and split the different colored channels (image → color → split channels)
3. For the hBMSCs and cancer cell channels do the following
    a. Filter the image (process → filters → median → radius of 3 pixels)
    b. Reduce the bit size of the image (image → type → select 8-bit)
    c. Threshold the images (image → adjust → auto threshold → select Huang2)
    d. Clean up pixel errors by (process → binary→ open) and (process → binary → close)

b. Create a selection of anything but the cellular areas by analyzing particles (analyze → analyze particles) and put in the following as settings:
    i. Size: 100 - infinity (pixels)
    ii. Circularity: 0.00-1.00
    iii. Show: overlay mask
    iv. Include holes: not ticked

    e. In the mask file that is then created make the selection (edit→ selection → create selection)

4. For the nuclei channel do the following
    a. Filter the image (process → filters → median → radius of 3 pixels)
    b. Reduce the bit size of the image (image → type → select 8-bit)

c. Threshold the images (image → adjust → auto threshold → select Huang2)
d. Clean up pixel errors by (process → binary→ open) and (process → binary → close)
e. Make filled and separate nuclei by (process → binary → fill holes) and (process → binary → watershed)
f. Create a nuclei image for counting by (analyze → analyze particles) and put in the following as settings:
   i. Size: 100 - infinity (pixels)
   ii. Circularity: 0.00-1.00
   iii. Show: count mask
   iv. Include holes: ticked
g. Duplicate the created mask file (image → duplicate)
h. Restore the selection of each of the cell types in one of the nuclei mask files and clear out nuclei that are not in the area of each cell type (edit → selection → clear)
i. Next count the nuclei
   i. (edit → selection → none)
   ii. (process → find maxima, prominence of 20)

1.3. Cellular quantities and ratios in bone metastasis spheroids, z-stacks

1. Make sure black background at options for binary is ticked (process → binary → options…)
2. Open the z-stack file and split the different colored channels (image → color → split channels)
3. Filter the image (process → filters → median → radius of 3 pixels). It will ask if you want to process all slides, click yes.
4. Threshold the z-stacks per cell type with the biovoxxel plugin
   a. Install the biovoxxel plugin (Biovoxxel.github.io)
   b. Threshold the z-stacks per color (plugin → biovoxxel 3D box → segmentation → threshold check (2D/3D), and set the following settings:
      i. Threshold library: CLIJ2
      ii. Histogram usage: full (default)
      iii. Contrast saturation: 0.00
      iv. Binary output style: tick 0/255
      v. Auto threshold (for cancer cells): Huang
      vi. Auto threshold (for hBMSCs): Li
5. Clean up pixel errors by (process → binary→ open) and (process → binary → close)
c. To remove other small speckles create a mask with (analyze → analyze particles) and put in the following as settings:
      iii. Size: 200 - infinity (pixels)
      iv. Circularity: 0.00-1.00
      v. Show: mask
      vi. Exclude edges: ticked
6. To get white objects on black background again change the LUT (LUT → grays)
7. Measure the volumes per cell type (plugins → 3DSuite → 3D Manager)
   a. First click on options (picture of tools), and select volume for measurement.
   b. Then select the mask image and click on 3D segmentation of the 3D manager. With threshold set to 1 and 225 (as it is already a binary image).
   c. Then change the image back to 8 bit (image → type → 8-bit) (only possible if you have less than 255 items)

d. Click on add image
e. Click on measure 3D
f. This gives you the list of the volumes per cell type (in pixels, thus recalculate it back to volumes with the known scale and step length of the z-stack)

1.4. Spatial organization in bone metastasis spheroids

1. Load image into FIJI and find the slice that is 25% into the spheroid, duplicate only this slice (image → duplicate)
2. Split the different colored channels (image → color → split channels)
3. Filter the images of the cells (process → filters → median → radius of 3 pixels)
4. Merge the channels of the hBMSCs and cancer cells (image → color → merge channels…), and duplicate the merged image (image → duplicate), one will be for the core selection, and the other for the periphery selection.
5. With one image find the selection of the whole spheroid, the core of the spheroid, and the periphery of the spheroid.
    a. Change LUT of both the green and red channels into grays (LUT → grays)
    b. Flatten image: (image → overlay → flatten)
    c. Change image back to 8 bit (image → type → 8 bit)
    d. Make sure black background at options for binary is ticked (process → binary → options…)
    e. Threshold the image (image → adjust → auto treshold → huang2)
    f. Clean up pixel errors by (process → binary→ open) and (process → binary → close)
    g. Fill up the spheroid with (process → binary → fill holes)
    h. Make a selection and add it to the ROI manager with analyzing particles (analyze → analyze particles), with the following settings:
        i. Size: 1000 - infinity (pixels)
        ii. Circularity: 0.00-1.00
        iii. Show: nothing
        iv. Include holes: ticked
        v. Add to manager: ticked
        vi. If necessary: check with the ROI manager which of the ROI is the whole spheroid and delete the others (if necessary rename the ROI into total)
    i. Now also find a selection for the core of the spheroid
        i. Set the measurements (analyze → set measurements → tick centroid and feret's diameter)
        ii. Go to the ROI manager and measure (click measure). This gives the x and y coordinates of the middle of the selection of the spheroid and the ferret diameter. (in µm)
            1. To get the same information in pixels make the following calculation: pixels = µm x scale.
                a. You can find the scale at (analyze → set scale) and look at distance in pixels.
        iii. The core selection can be made by running a macro (plugin → new → macro), all in pixels
            1. Write: makeOval(x, y, width, height)
                a. x = (x found in measurements) – ½ width
                b. y = (y found in measurements) – ½ width
                c. width = height = ½ (ferret diameter)

2. Click on Run
3. Save this selection into ROI manager as well by clicking on add in the ROI manager

j. To create the selection of the periphery of the spheroid, select both the ROI of the total spheroid and the core and in the ROI manager click on (more →XOR), then click on add.

6. Now go back to the other image that was duplicated
   a. Image → color → stack to RGB
   b. Go to ROI manager and do the following for both the core and periphery selection
   c. Run the plugin color pixel counter (plugin → color pixel counter)
      i. Select either green or red
      ii. Only tick display color percentage
      iii. For green minimum intensity values: 20
      iv. For red minimum intensity values: 5

1.5. Cellular quantities and ratios in bone metastasis spheroids, slices

1. Open images, and delete any pixels that are not part of the main spheroid that needs to be measured in that slice, collect all cleaned up files in one folder with recognizable names.
2. Put the following text as a macro (process→batch→macro). Select the input folder with the slices that need to be measured and do not select any output folder. The data will be summarized in one go.

```
name = getTitle();

rename("1");
run("Split Channels");
selectWindow("C2-1");
close();

selectWindow("C1-1");
rename(name+"green");
run("Median...", "radius=3");
run("Auto Threshold", "method=Default white");
run("Open");
run("Close-");
run("Analyze Particles...", "size=200-Infinity pixel show=Overlay display clear summarize");
selectWindow(name+"green");
close();

selectWindow("C3-1");
rename(name+"red");
run("Median...", "radius=3");
run("Auto Threshold", "method=Default white");
run("Open");
run("Close-");
run("Analyze Particles...", "size=200-Infinity pixel show=Overlay display clear summarize");
selectWindow(name+"red");
close();
```

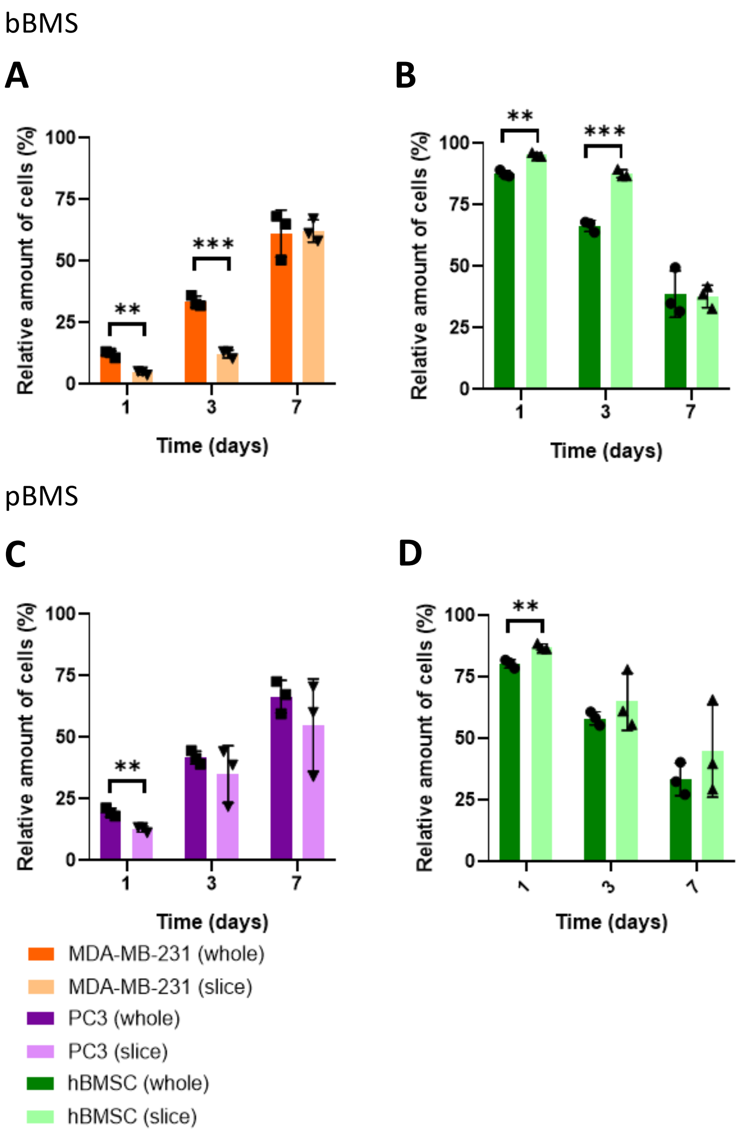

Figure S1: *Comparison of relative quantity of cells in entire Z-stacks versus relative quantity of cells in Z-stack slices at 25% depth of BMS composed of either MDA-MB-231 cells (A) and hBMSCs (B), or PC3 cells (C) and hBMSCs (D) at days 1, 3, and 7 in culture show similar trends independent of the chosen method of measurement. Quantitative data are based on (n=3) spheroids and were statistically analyzed using independent t-test on each point in time for each cell type between whole and slice measurements; **p<0.01, ***p<0.001.*

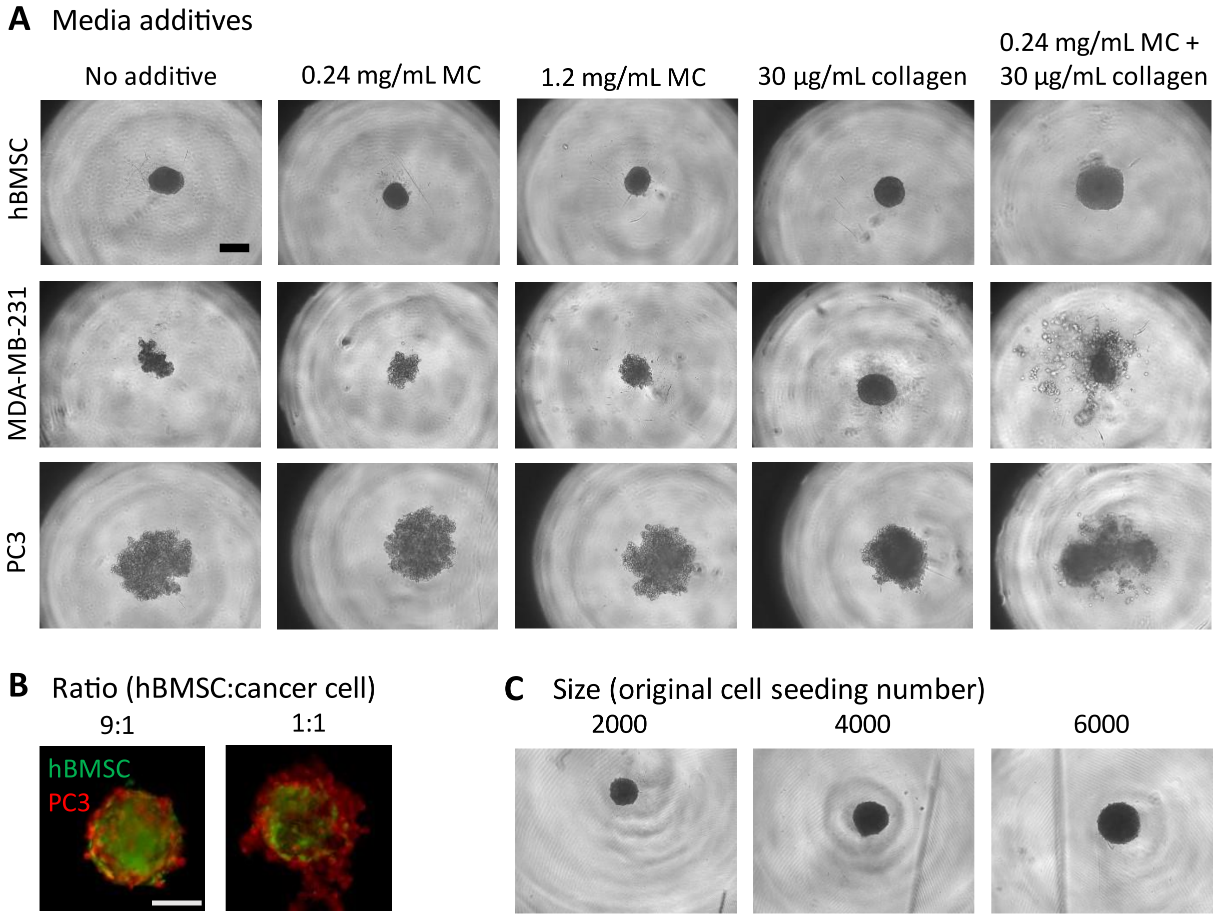

Figure S2: *Development of the 3D co-culture model for bone metastases by exploring various culture conditions for stable spheroid production.* *Brightfield images of monocultured spheroids (A),) were recorded at day 3 in culture with various media supplements to determine the right substance and concentration. To find the proper ratio of hBMSCs and cancer cells, cells were seeded at hBMSC: PC3 ratios of 9:1 and 1:1 ratio and subsequently imaged with laser scanning confocal microscopy (B). Finally, the cell seeding density for stable spheroid production was determined using brightfield imaging (C) with bone metastatic spheroids composed hBMSCs and PC3 cells (hBMSC:PC3 ratio of (9:1) at 2000, 4000, and 6000 cells per spheroid. Scale bar represents 300 µm in the brightfield images, and 100 µm in the fluorescence images.*

Table S1: Circularity values of spheroids (n = 4) with various media supplements.

	No additive	0.24 mg/mL MC	1.2 mg/mL MC	30 µg/mL collagen	0.24 mg/mL MC + 30 µg/mL collagen
hBMSC	0.87	0.86	0.812	**0.91**	**0.91**
PC3	0.46	**0.52**	0.41	0.43	X
MDA-MB-231	0.39	0.38	0.58	**0.89**	X

X: no image quantification as no proper spheroids were formed

Table S2: Circularity values of spheroids (n = 4) seeded with different total cell numbers.

	2000	4000	6000
hBMSC +MDA	**0.87**	*	*
hBMSC + PC3	**0.79**	0.71	0.73

* no data

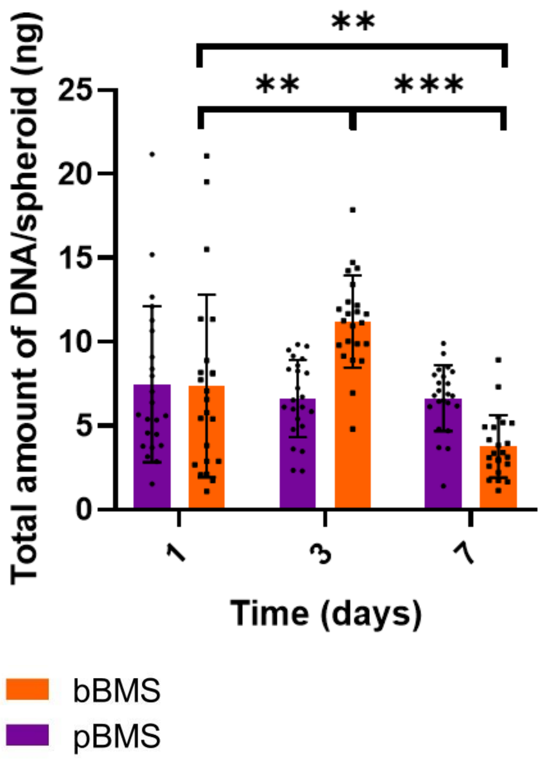

Figure S3: *Total DNA content of bBMSs and pBMSs up to 7 days of culture. Quantitative data are based on (n=23) spheroids and were statistically analyzed using an one-way ANOVA for the bBMSs and pBMSs; **p<0.01, ***p<0.001.*

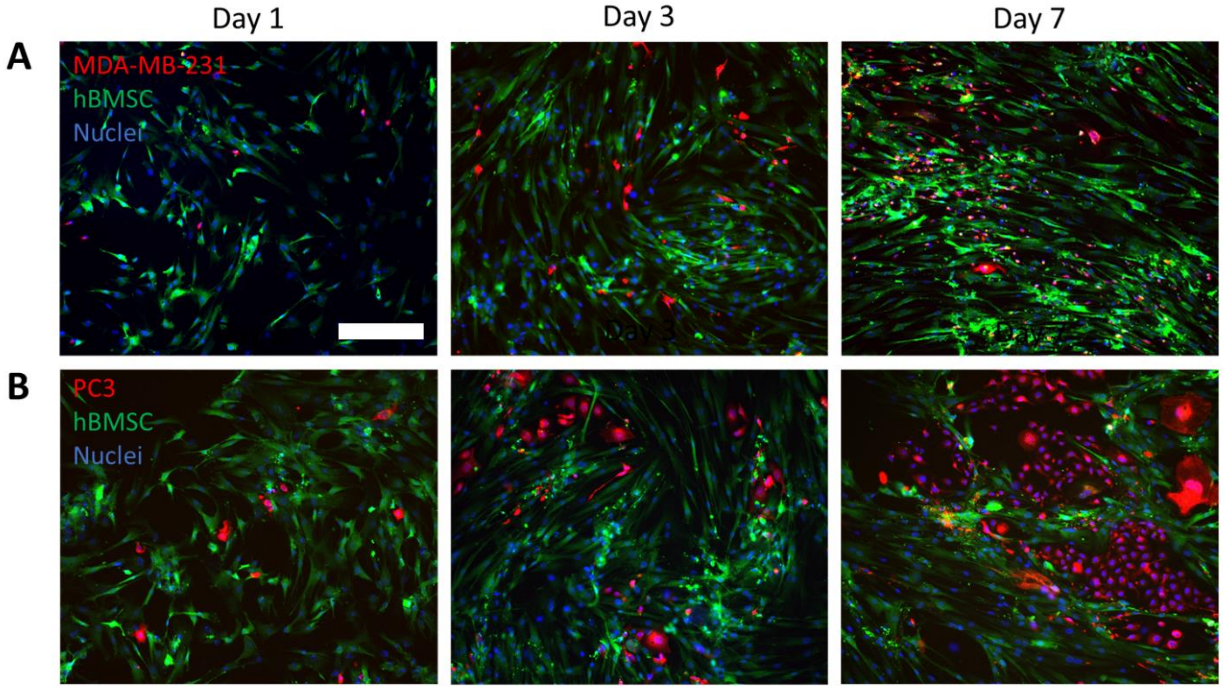

Figure S4: *Fluorescence images of 2D co-cultures with either breast cancer (A) or prostate cancer cells (B) with hBMSCs. hBMSCs are colored green, cancer cells red, and nuclei blue. Scalebar represents 100 µm.*

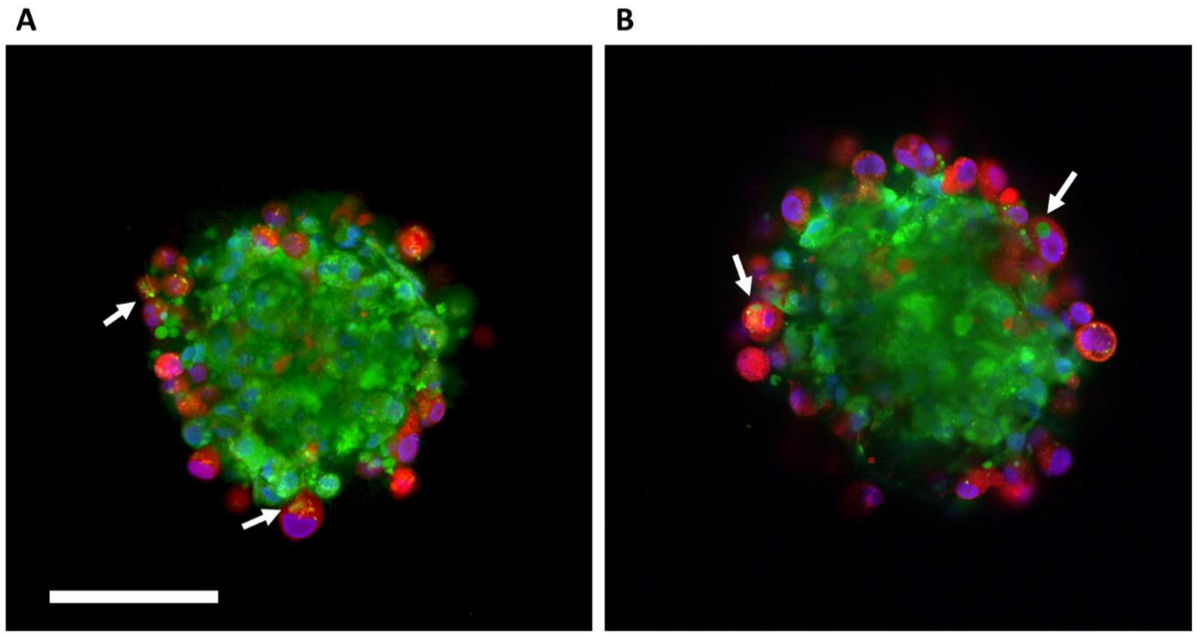

__Figure S5:__ Fluorescence images of bBMS (A) and pBMS (B) at day 3 in culture, with white arrows pointing to the green colored vacuoles in the cancer cells. Scalebar represents 300 µm). hBMSCs are colored green, cancer cells red, nuclei blue.

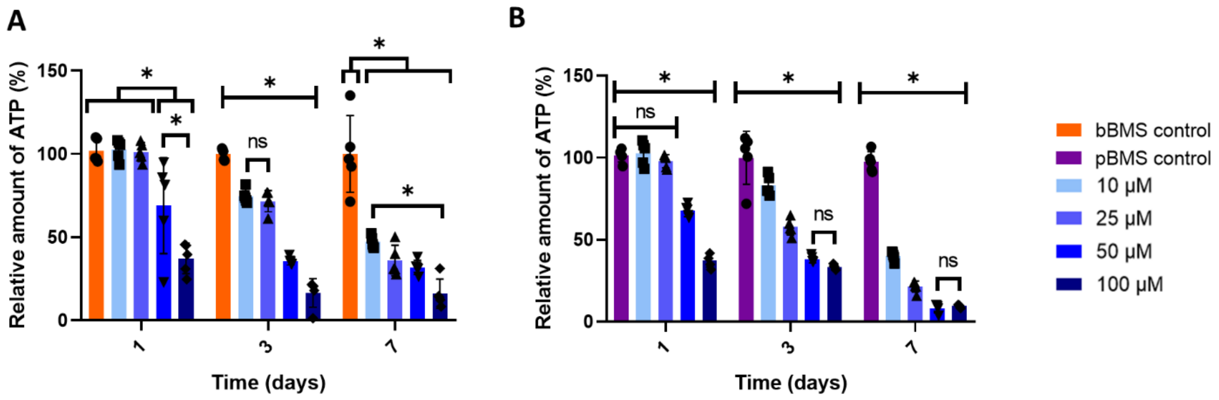

*__Figure S6:__ Relative amount of total ATP content in (A) bBMSs and (B) pBMSs when treated with various concentrations of cisplatin. Quantitative data are based on spheroids (n=5) and were statistically analyzed using a one-way ANOVA on each time point; *p<0.05.*
